# Gold film-catalysed benzannulation by Microwave-Assisted, Continuous Flow Organic Synthesis (MACOS)

**DOI:** 10.3762/bjoc.5.35

**Published:** 2009-07-21

**Authors:** Gjergji Shore, Michael Tsimerman, Michael G Organ

**Affiliations:** 1 Department of Chemistry, York University, 4700 Keele Street, Toronto, ON, Canada M3J 1P3

**Keywords:** benzannulation, flow synthesis, gold catalysis, microwave, thin metal film

## Abstract

Methodology has been developed for laying down a thin gold-on-silver film on the inner surface of glass capillaries for the purpose of catalysing benzannulation reactions. The cycloaddition precursors are flowed through these capillaries while the metal film is being heated to high temperatures using microwave irradiation. The transformation can be optimized rapidly, tolerates a wide number of functional groups, is highly regioselective, and proceeds in good to excellent conversion.

## Introduction

Microwave-assisted organic synthesis (MAOS) has had a significant impact on organic and medicinal chemistry by dramatically shortening reaction times, producing cleaner product mixtures, and making high-energy transformations routine that might otherwise be avoided [1–2]. Within the last five years, microwave technology has also been applied to reactions performed in a flowed format [3–4]. Flowed chemical synthesis offers numerous advantages over traditional batch-reactor technology [5–24]. Independent inlet streams allow reactive intermediates to be kept separate until brought together in miniscule amounts to react immediately; this rapidly depletes the starting materials and continuously physically moves the product away from the infusing stream. In batch reactors, product molecules form in the presence of a vast excess of starting materials that can lead to significant byproduct formation. Further, a moving synthesis platform allows for in-line analysis and instantaneous changes to reaction conditions for process optimization that can be automated readily. To gain the full advantage of working in flow, reactions should proceed very rapidly and ideally reach completion during the time in which the reactants reside in the flow tube. Microwave heating has been used to drive a wide variety of reactions to high levels of completion in a flowed format [5–24].

We have demonstrated that thin-metal films on the walls of narrow tubes can impart tremendous rate accelerations on flowed reactions that are being simultaneously microwaved [25–28]. These accelerations can be a result of direct catalysis by the film itself, the tremendous temperatures attainable by microwaved metal films, or a combination of the two. In cross coupling processes, the film has been demonstrated itself to be capable of catalyzing Suzuki–Miyaura and Heck reactions, i.e., Pd-thin films promote these transformations without any additional catalyst being added to the reactant stream(s) that enter the flow tube [28]. Without microwave irradiation, the above-described cross-coupling reactions did not proceed indicating that there is not only a catalytic effect, but also a pivotal heating effect supplied by the film. More recently we have shown that a gold film in the same flow reactor is highly effective for hydrosilylation reactions [25] and we were interested both to expand the use of gold films in synthesis using MACOS and in exploring complex, multi-step catalytic processes in flow.

**Figure 1:**
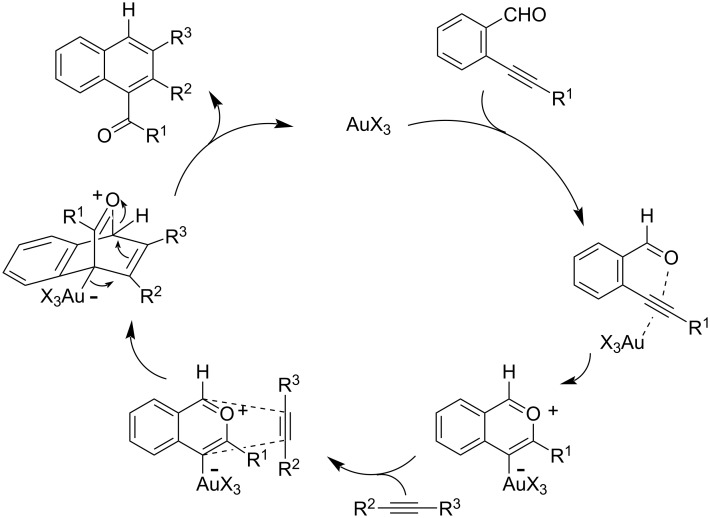
Mechanism of Au(III)-catalyzed benzannulation between aromatic carbonyls and alkynes.

The benzannulation reaction between aromatic carbonyls and alkynes has received increasing attention since 2002 [29–30]. This transformation has been shown to be promoted by Lewis acids, copper complexes [31] and various gold species, including Au(I)X, Au(III)X_3_, [32–34] and Au nanoparticles dispersed on different supports. For Au(III) catalysts, the reaction has been proposed to proceed via the formation of no less than four organogold intermediates and/or complexes (Figure 1).

Further, it has been shown computationally by Straub that Au(I) and Au(III) can perform both this and related catalytic cycles with similar energy profiles [35]. This blurs the distinction of the two pathways and raises the possibility that the actual active species in these transformations could be either species, providing that the possibility exists for the starting Au complex to be oxidized or reduced under a specific set of reaction conditions. Given the complexity of this process, we thought that benzannulation would be an ideal transformation to investigate the use of gold films for MACOS.

## Results and Discussion

**Figure 2:**
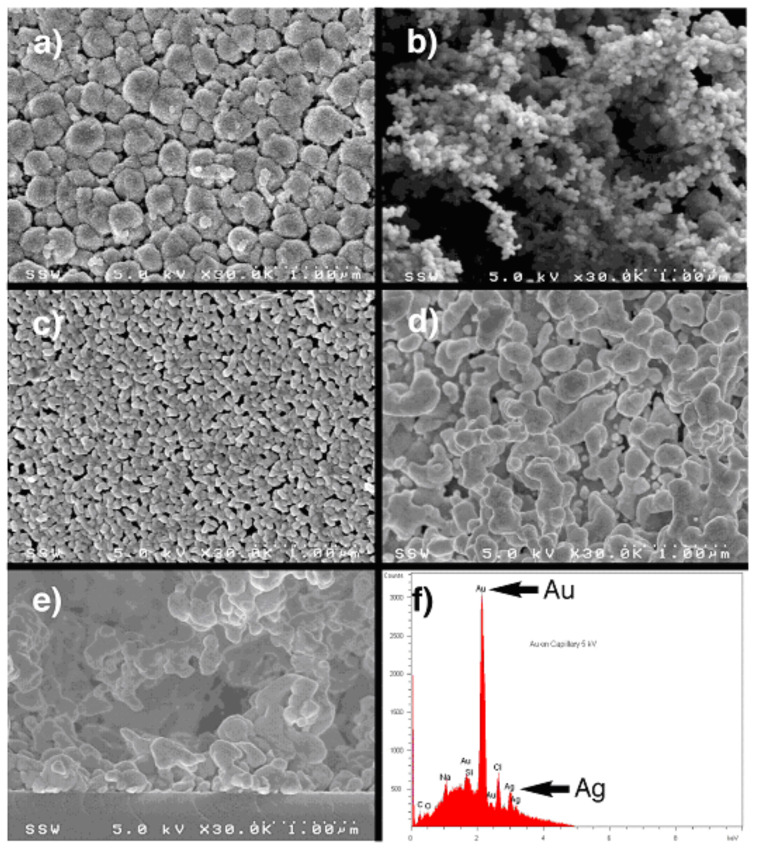
X-ray analysis of the metal films used in this benzannulation study. Panels a–e are scanning-electron micrographs (SEM) of all films taken at 30,000 × magnification: a) Cu, b) Pd, c) Ag, d) Au layered on a base film of Ag. Panel e shows the side view of the Au film attached to the glass capillary wall (bottom of image), between which is sandwiched a barely-visible thin Ag coating. Panel f is the energy-dispersive X-ray (EDX) spectrum of the Au film from panel d; note the small level of Ag that is present that helps to anchor the Au to the surface of the glass.

Our investigation started with an assessment of different types of metal films for their utility in benzannulation. The morphology of Cu, Pd, Ag, and Au films can be compared in the scanning electron microscopy (SEM) images in Figure 2. The films are generally prepared in a reducing environment (see Experimental), thus they are expected to be largely M(0), which has been confirmed by energy-dispersive X-ray (EDX) analysis of the films (e.g., see panel f in Figure 2).

However, film preparation was not conducted under a strictly inert environment, thus oxygen and/or water in the air could play a role in the film’s composition leading to the formation metal oxides, for example, on the surface. Palladium films had the greatest porosity (panel b) while silver films (panel c) were the most dense and uniform. Gold films showed poor adhesion to the glass and poor performance when subjected to the rigors of MACOS. However, these properties were dramatically improved when the same gold film was laid down on a transparently-thin silver layer that coated the capillary wall. With these metal-film-coated capillaries in hand, benzannulation was investigated using the substrates shown in Table 1.

**Table 1:** Optimization Studies for Benzannulation Reaction using MACOS.

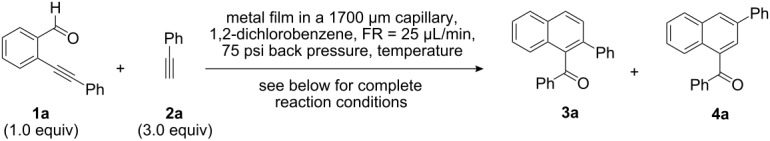						
Entry	Film	Heat Source	Temp. (°C)	Percent Conversion^a^	Percent Yield^b^	**3a** : **4a**

1	Cu	microwave	240	5	4	ND
2	Pd	microwave	240	0	0	–
3	Ag	microwave	240	0	0	–
4	Au on Au	microwave	220	75	ND	
5	Au on Ag	microwave	240	90	78	3 : 1
6	Au on Ag	microwave	190	68	ND	ND
7	Au on Ag	oil bath^c^	190	14	ND	ND

^a^Percent conversion was determined by ^1^H NMR spectroscopy by comparing the ratio of product peaks (**3** and/or **4**) to the starting aldehyde (**1**) from aliquots taken directly from the eluent stream. ^b^Percent yield was determined by collecting a specific volume of eluent that contained a known amount of starting materials, and purifying the material by silica gel flash chromatography. ^c^The metal-film-coated capillary was left in the bath for 20 min to ensure that the film was at temperature prior to flowing the reaction mixture through it.

This reaction is reported most widely using homogeneous and heterogeneous gold catalysts, and indeed we found that a pure Au film provided good conversion for a short period (Table 1, entry 4), although the film had a short lifespan. Films of palladium (entry 2) and silver (entry 3) showed no conversion of starting materials at all. Surprisingly, copper, which is reported to be a suitable catalyst for benzannulation, could not be optimized beyond the formation of only trace amounts of product (entry 1). We experimented with the use of bimetallic films and found that optimal film performance was achieved by laying down a porous gold film on top of a thin silver mirror. When run at a high temperature (240 °C, entry 5), as read by the IR sensor in the Biotage Initiator microwave, excellent conversion was achieved that diminished as the temperature was reduced (e.g., 190 °C, entry 6). It is noteworthy that under batch conditions, these reactions are reported to require up to 6 h to obtain similar conversion levels [7] to those obtained in less than 60 s using MACOS, which is the residence time of any reactant plug in the flow reactor.

To probe any special effect brought about by the microwave in this transformation, the identical transformation in entry 6 was performed using an oil bath to achieve the same temperature (i.e., 190 °C, entry 7) and the conversion was dramatically reduced from 68 to 14 percent. It is possible that superior microwave conversion is the result of localized ‘hot spots’ in the film that are potentially well above the bulk temperature recorded by the IR sensor when microwave irradiation is used. It has been proposed that steps or angles in the surface of the metal film can serve as an antenna resulting in higher levels of microwave absorption in those areas causing higher than normal temperatures. In an oil bath (or oven), only the bulk temperature of the external heating source can be achieved in the film. If this is the case, and localized areas of potentially extreme temperature are required for optimal reaction performance, this would be preferable to heating the entire film to these higher temperatures, which would lead to faster film and product decomposition.

With optimized conditions in hand, we set out to examine the substrate scope of this reaction using capillaries lined with the gold-on-silver films (Table 2).

**Table 2:** Benzannulation reactions with Au on Ag-coated capillaries using MACOS.

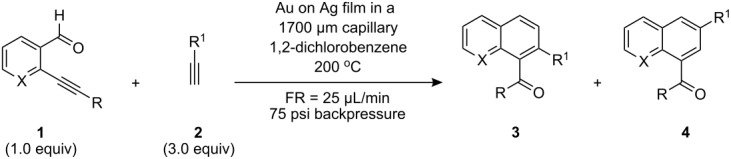						
Entry	R–	–X–	R^1^–	Percent Conversion^a^	Percent Yield^b^	**3** : **4**

**a**	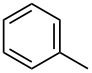	–CH–	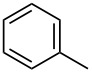	90	78	75 : 25
**b**	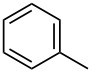	–CH–	(CH_3_)_3_Si–	75	62	**4** only
**c**	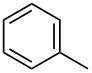	–CH–	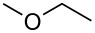	62	52	58 : 42
**d**	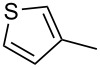	–CH–	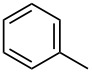	76	62	**3** only
**e**	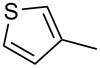	–CH–	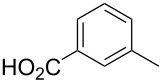	72	60	**4** only
**f**	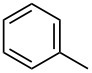	–N–	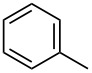	78	64	70 : 30
**g**	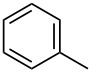	–N–	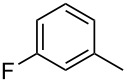	68	54	**4** only
**h**	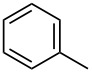	–N–	(CH_3_)_3_Si–	65	58	**4** only
**i**	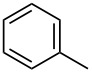	–N–	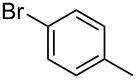	65	52	**4** only
**j**	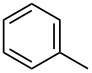	–N–	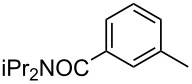	50	40	**4** only

^a^Percent conversion was determined by ^1^H NMR spectroscopy by comparing the ratio of product peaks (**3** and/or **4**) to the starting aldehyde (**1**) from aliquots taken directly from the eluent stream. ^b^Percent yield was determined by collecting a specific volume of eluent that contains a known amount of starting materials, and purifying the material by silica gel flash chromatography.

The reaction was applicable to the hydrocarbon starting materials tried (e.g., entries **a**, **b**, **c**) and was also useful for the preparation of heterocycle-containing molecules such as pyridines and thiophenes (e.g., entries **e**–**j**). A survey of different functional groups revealed a broad tolerance including silyl groups (entries **b** and **h**), halides (entries **g** and **i**), ethers, (entry **c**), amides (entry **j**), and even free carboxylic acids (entry **e**). In all cases where reactions did not fully complete, benzaldehyde (**1**) accounted for the mass balance; crude ^1^H NMR spectra were very clean showing only residual **1**, product and trace by products. In most cases, the reactions displayed a high level of regioselectivity.

All of the above runs were conducted on approximately 700 μL of an infusing starting material solution (35 min per run), which, after purification, generated ~70–80 mg of final product. Such quantities are ample for evaluation in biological screens. However, larger quantities of a compound can be required, so using the substrates in Table 2, entry **a**, a larger-scale benzannulation was performed. To improve efficiency and to reduce solvent consumption (and hence waste production) the concentration of both starting materials was tripled. After running the reactor for 90 min, three quarters of a gram of product (**3a** and **4a**, 3:1) were collected. Under these conditions the concentration of alkyne **2a** was 4.5 M, demonstrating that MACOS can easily handle concentrations that are far above the typical levels (0.1–0.5 M) commonly used in conventionally-heated batch reactors.

The lifetime and durability of the film under the optimized reaction conditions was examined. The transformation in Table 2, entry **a** was followed over the course of 2 h; reaction performance is optimal at the beginning and slowly, but steadily erodes. Visibly, the film darkens over time, which we attribute to small amounts of starting materials and/or products that char over time on the surface of the hot film; this may lead to the blockage of reaction sites. Additionally, the thin-walled capillary glass softened and in some cases failed after 90 min of exposure to the hot film. While bulk manufacturing of very large quantities is not yet attainable with the current reactor design, the device is very suitable for preparing library collections of 50–750 mg of product for evaluation in medicinal chemistry or materials science applications.

## Conclusion

Glass capillaries internally-lined with thin-metal films of gold, copper, silver, and gold-on-silver were prepared and evaluated for their catalytic activity in the microwave-assisted, continuous flow benzannulation of aryl, alkyl and silylalkynes with alkynylbenzaldehydes. Only the gold-on-silver films possessed both the high level of catalytic activity and suitable physical robustness to prepare quantities of product suitable, for example, for biological or material science evaluation purposes (up to ~700 mg). The reaction showed wide functional group tolerance and good to excellent regioselectivity in the cycloaddition. We are now evaluating new materials to replace the glass tube with the plan of making the process more sustainable.

## Experimental

### Microwave irradiation experiments

All MACOS experiments were performed in 1700 μm (ID) borosilicate capillaries, using a single mode Biotage Smith Creator Synthesizer, operating at a frequency of 2.45 GHz with irradiation power from 0 to 300 W. The capillary was fed reactants from Hamilton gastight syringes attached to a Harvard 22 syringe pump pre-set to the desired flow rate. The system was connected to a sealed collection vial, where a pressurized air line (75 psi) was attached to create backpressure. The temperatures reported were measured off the surface of the capillaries by the IR sensor built into the microwave chamber. All reagents and solvents were purchased from commercial sources and used without additional purification. Column chromatography purifications were carried out using the flash technique on silica gel 60 (200–400 mesh). ^1^H NMR spectroscopy was run using a Bruker Advance 400 MHz instrument and all spectra were calibrated to the signal from the residual proton of the deuterated chloroform solvent (7.26 ppm); ^13^C NMR spectra were calibrated to the middle carbon signal of the triplet for deuterated chloroform (77.00 ppm).

### General procedure for the benzannulation reactions by MACOS

A stock solution containing the acetylenic aldehyde (0.5 mmol, 1.0 equiv.) and alkyne (1.5 mmol, 3.0 equiv) in 0.7–0.8 mL 1,2-dichloro benzene (total mixture volume is 1.0 mL) was prepared and loaded into a Hamilton gastight syringe. The tubing was primed with 1,2-dichloro benzene and the syringe was connected to the reactor system with the aid of Microtight fittings after which it was placed in a Harvard 22 syringe pump that was set to deliver 20 μL/min. The single mode microwave was programmed to heat constantly; the power level was controled manually so as to keep the temperature constant at the specified levels. The effluent from the reactor was fed into a sealed vial and analyzed directly by ^1^H NMR spectroscopy immediately after the reaction. Typically, 0.7–0.8 mL of the crude reaction mixture was collected and the product was purified by silica gel column chromatography.

### General procedure for creating the gold-on-silver film coating inside of 1700 micron (ID) capillaries

Tollens’ reagent (0.5 mL) was mixed with 0.5 mL of a 5% D-glucose solution into a 2 mL vial. The 1700 μm capillaries (ID) were filled with this mixture, capped at both ends and left to develop at rt. After the Ag coating was fully developed (15 min), the capillaries were rinsed with acetone and placed inside a muffle furnace for calcination at 500 °C (3 × 1 min).

The gold-coating solution was prepared by mixing a 0.4 mmol/mL aqueous solution of AuCl_3_ (0.5 mL) with a 2% aqueous solution of sodium citrate (0.5 mL). The Ag-lined capillaries were filled with the mixture, capped at both ends, and left to develop at rt for an additional 30 min. After emptying the capillaries and rinsing them with acetone, they were calcinated at 500°C (3 × 1min) before use in MACOS.

Tollen’s reagent was prepared as follows: 2.0 mL of 4M NaOH was added drop-wise to 20 mL of a 3% AgNO_3_ solution, forming a gray precipitate that was titrated with a 4M solution of NH_4_OH until the solution became clear.

### General procedure for synthesis of benzaldehydes [36]


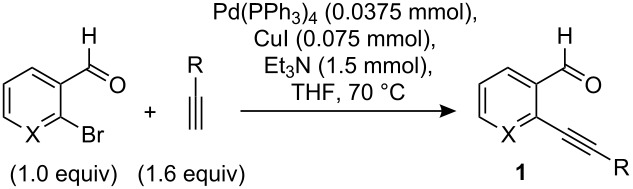


A 10 mL round-bottom flask was charged with Pd(PPh_3_)_4_ (0.0375 mmol), CuI (0.075 mmol), and purged with argon. To it were added consecutively: THF (6 mL), Et_3_N (1.5 mmol), the aryl bromide (0.75 mmol), and the alkyne (0.9 mmol). The solution was then heated to 70 °C and stirred for 6–13 h (until the reaction was judged complete by tlc analysis). At the end of the reaction, the mixture was diluted with H_2_O (10 mL) and extracted with ethyl acetate (3×). The combined organic layers were dried over anhydrous Na_2_SO_4_, concentrated, the residue dry loaded onto silica gel and purified by column chromatography.


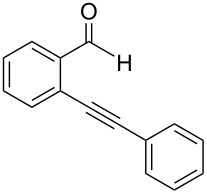


**2-(2-Phenylethynyl)benzaldehyde** (**1a**). Following the general procedure for the synthesis of benzaldehydes, column chromatography (10% diethyl ether in pentane) provided 141 mg of product as a pale-brown oil (91%, yield). ^1^H NMR (300 MHz, CDCl_3_): δ 10.67 (s, 1H), 7.96 (d, *J* = 7.8 Hz, 1H), 7.63 (t, *J* = 4.5 Hz, 1H), 7.57 (m, 3H), 7.44 (t, *J* = 7.2 Hz, 1H), 7.41–7.37 (m, 3H). ^13^C NMR (75 MHz, CDCl_3_): δ 191.6, 135.8, 133.8, 133.2, 131.7, 129.1, 128.6, 128.5, 127.3, 126.9, 122.4, 96.4, 85.0. Spectra matched that found in the literature [37].


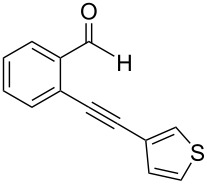


**2-[2-(Thiophen-3-yl)ethynyl]benzaldehyde** (**1d**). Following the general procedure for the synthesis of benzaldehydes, column chromatography (10% diethyl ether in pentane) provided 142 mg of the product as a pale-yellow oil (89%, yield). ^1^H NMR (300 MHz, CDCl_3_): δ 10.63 (s, 1H), 7.95 (d, *J* = 8.1 Hz, 1H), 7.66–7.51 (m, 3H), 7.43 (t, *J* = 7.8 Hz, 1H), 7.34 (dd, *J* = 5.1, 3.0 Hz, 1H), 7.23 (dd, *J* = 5.1, 1.2 Hz, 1H). ^13^C NMR (75 MHz, CDCl_3_): δ 191.7, 135.8, 133.8, 133.1, 129.7 (two carbons overlap), 128.5, 127.3, 126.9, 125.8, 121.4, 91.5, 84.6. HRMS Calcd. for C_13_H_8_OS: 212.0296; found: 212.0302: Anal. Calcd. for C_13_H_8_OS: C, 73.56; H, 3.80; found C, 73.82; H, 3.57.


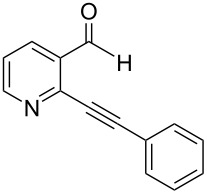


**2-(2-Phenylethynyl)nicotinaldehyde** (**1f**). Following the general procedure for the synthesis of benzaldehydes, column chromatography (30% diethyl ether in pentane) provided 145 mg of product as a dark-yellow solid (93%, yield). Mp = 91–92 °C. ^1^H NMR (400 MHz, CDCl_3_): δ 10.70 (s, 1H), 8.85 (d, *J* = 3.6 Hz, 1H), 8.24 (d, *J* = 7.6 Hz, 1H), 7.67 (d, *J* = 6.8 Hz, 2H), 7.55–7.30 (m, 4H). ^13^C NMR (100 MHz, CDCl_3_): δ 190.8, 154.5, 146.1, 134.9, 132.2, 131.8, 129.9, 128.6, 123.2, 121.2, 96.1, 84.6. HRMS Calcd. for C_14_H_9_NO 207.0684; found 207.0685. Anal. Calcd. for C_14_H_9_NO: C, 81.14; H, 4.38; found C, 81.43; H, 4.60. Spectra matched that found in the literature [38].


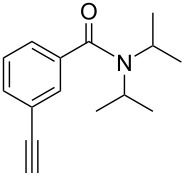


**3-Ethynyl-*N,N*-diisopropylbenzamide** (**2j**). A 25 mL round-bottom flask was purged with argon, cooled to 0 °C, and 2.4 mL of dry THF and 157 μL (1.8 mmol) of oxalyl chloride were added. After 20 min., a cooled (0 °C) solution of 3-ethynyl-benzoic acid (1.71 mmol, 250 mg) in 6 mL of dry THF was added over 10 min. When the addition of acid was complete, 3 drops of DMF were added to the mixture and bubbling was observed. After stirring for 30 min. at 0 °C, the solution was stirred an additional 30 min. at rt, and then cooled back to 0 °C. Into a separate 25 mL round-bottom flask at 0 °C were added: (iPr)_2_NH (1.8 mmol, 260 μL), Et_3_N (1.8 mmol, 250 μL), and 2 mL of dry THF. The contents of the original flask were then added to this flask where upon fuming was observed and a yellow precipitate began to form. The reaction was warmed to rt, stirred for 16 h, and quenched by the addition of 10 mL of water. After stirring for 10 min., the contents were then transferred to a separatory funnel and ethyl acetate was added. The separated organic layer was washed with sat NaHCO_3_ and the aqueous layer was back-extracted twice with ethyl acetate. The combined organic layers were washed with brine, dried over anhydrous Na_2_SO_4_, and filtered. Following solvent removal *in vacuo*, the yellow solid residue was dry-loaded onto silica gel and purified by column chromatography using a gradient elution (5% ethyl acetate in pentane to 15% ethyl acetate in pentane, R_f_ = 0.5) to give 390 mg product as a white solid (99% yield). Mp = 107–108 °C. ^1^H NMR (400 MHz, CDCl_3_): δ 7.49 (d, *J* = 7.6 Hz, 1H), 7.43 (s, 1H), 7.36 (t, *J* = 8.4 Hz, 1H), 7.28 (d, *J* = 8.4 Hz, 1H), 7.32 (d, 2H), 3,09 (s, 1H), 1.33 (d, 12H). ^13^C NMR (150 MHz, CDCl_3_, 10 °C): δ 169.8, 139.0, 132.2, 129.1, 128.6, 125.9, 122.4, 82.9, 77.9, 51.0, 45.9, 20.7, 20.6. HRMS Calcd. for C_15_H_19_NO: 229.1467; found: 229.1466. Anal. Calcd. for C_15_H_19_NO: C, 78.56; H, 8.35; found C, 78.61; H, 8.71.


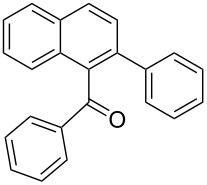


**Phenyl-(2-phenylnaphthalen-1-yl)-methanone** (**3a**). Following the general MACOS benzannulation protocol, 2-(2-phenylethynyl)-benzaldehyde (**1a**) and phenylacetylene were reacted and 700 μL of the crude reaction mixture were collected. Purification by flash chromatography (15% ethyl acetate in hexane) afforded 62 mg of **3a** and 20 mg of **4a** as yellow oils (78% combined yield, minor isomer reported below). ^1^H NMR (400 MHz, CDCl_3_): δ 8.2 (d, *J* = 8.2 Hz, 1H), 7.95 (d, *J* = 8.3 Hz, 1H), 7.78 (d, *J* = 8.1 Hz, 1H), 7.65 (m, 2H), 7.58 (d, *J* = 8.3 Hz, 1H), 7.35–7.52 (m, 5H), 7.15–7.30 (m, 5H). ^13^C NMR (100 MHz, CDCl_3_): δ 199.8, 140.4, 137.9, 137.6, 137.2, 133.4, 132.5, 130.5, 129.5, 129.4, 129.3, 128.3, 128.1, 128.0, 127.6, 127.2, 127.0, 126.4, 125.5. Spectra matched that found in the literature [39].


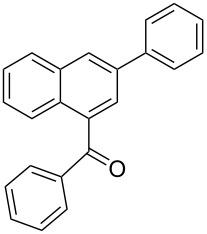


**Phenyl-(3-phenylnaphthalen-1-yl)-methanone** (**4a**). ^1^H NMR (400 MHz, CDCl_3_) δ 8.15 (d, *J* = 1.1 Hz, 1H), 7.98 (d, *J* = 8.2 Hz, 1H), 7. 90 (d, *J* = 8.0 Hz, 1H), 7.80 (d, *J* = 8.0 Hz, 2H), 7.72 (d, *J* = 1.1 Hz, 1H), 7.58 (d, *J* = 8.0 Hz, 2H), 7.50 (tt, *J* = 7.4, 1.0 Hz, 1H), 7.37–7.47 (m, 6H), 7.30 (tt, *J* = 7.4, 1.0 Hz, 1H). ^13^C NMR (100 MHz, CDCl_3_): δ 197.7, 139.9, 138.2, 137.2, 137.0, 134.2, 133.4, 130.5, 130.1, 129.0, 128.8, 128.6, 128.3, 127.8, 127.2, 127.0, 126.9, 126.7, 125.7. Spectra matched that found in the literature [40].


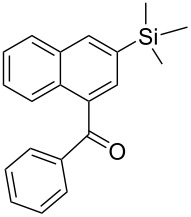


**Phenyl-(3-trimethylsilanyl-naphthalen-1-yl)-methanone** (**4b**). Following the general MACOS benzannulation protocol, 2-(2-phenylethynyl)-benzaldehyde (**1a**) and TMS-acetylene were reacted and 740 μL of the crude reaction mixture were collected. Purification by flash chromatography (15% ethyl acetate in hexane) afforded 68.9 mg of **4b** as a white solid (62% yield). Mp = 86–87 °C (lit. [34]: 88 °C). ^1^H NMR (400 MHz, CDCl_3_): δ 8.17 (d, *J* = 1.1 Hz, 1H), 8.05 (d, *J* = 8.2 Hz, 1H), 7.85–7.95 (m, 3H), 7.68 (d, *J* = 1.1Hz, 1H), 7.45–7.60 (m, 5H), 0.35 (s, 9H). ^13^C NMR (100 MHz, CDCl_3_): δ 198.5, 138.3, 136.8, 136.5, 135.5, 133.3, 133.0, 131.6, 131.1, 130.5, 128.5, 128.3, 127.4, 126.4, 125.6, −1.20. Spectra matched that found in the literature [34].


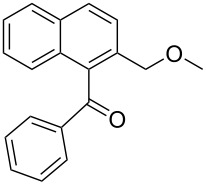


**[2-(Methoxymethyl)naphthalen-1-yl]-phenylmethanone** (**3c**). Following the general MACOS benzannulation protocol, 2-(2-phenylethynyl)-benzaldehyde (**1a**) and methyl propargyl ether were reacted and 815 μL of the crude reaction mixture were collected. Purification by flash chromatography (15% ethyl acetate in pentane) afforded 58.0 mg of **3c** and **4c** isomers as colourless oils (52% combined yield, 58:42, respectively).

(**3c**) ^1^H NMR (400 MHz, CDCl_3_): δ 7.98 (d, *J* = 8.1 Hz, 1H), 7.92 (d, *J* = 8.1 Hz, 1H), 7.84 (d, *J* = 8.1Hz, 2H), 7.53–7.66 (m, 3H), 7.37–7.52 (m, 4H), 4.50 (s, 2H), 3.23 (s, 3H). ^13^C NMR (100 MHz, CDCl_3_): δ 199.2, 137.8, 135.7, 133.7, 133.3, 132.8, 130.4, 129.6, 129.4, 128.7, 128.2, 126.8, 126.2, 125.5, 125.4, 72.1, 58.3. Anal. Calcd. for C_19_H_16_O_2_: C, 82.58; H, 5.84; found, C, 82.34, H, 5.60. HRMS calcd. for C_19_H_16_O_2_: 276.1150; found 276.1154.


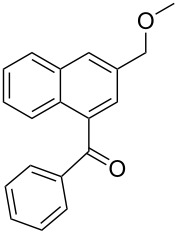


**[3-(Methoxymethyl)naphthalen-1-yl]-phenylmethanone** (**4c**). ^1^H NMR (400 MHz, CDCl_3_): δ 8.06 (d, *J* = 8.1Hz, 1H), 7.98 (d, *J* = 1.1 Hz, 1H), 7.93 (d, *J* = 8.1 Hz, 1H), 7.89 (d, *J* = 8.1 Hz, 2H), 7.10 (t, *J* = 7.1 Hz, 1H), 7.58 (d, *J* = 1.1 Hz, 1H), 7.45–7.56 (m, 4H), 4.66 (s, 2H), 3.46 (s, 3H). ^13^C NMR (100 MHz, CDCl_3_): δ 197.9, 138.2, 136.7, 134.4, 133.7, 133.3, 130.5, 130.4, 129.4, 128.5, 128.3, 127.3, 127.1, 126.7, 125.6, 74.2, 58.3. Anal. Calcd. for C_19_H_16_O_2_: C, 82.58; H, 5.84; found C, 82.39; H, 5.62. HRMS calcd. for C_19_H_16_O_2_: 276.1150; found 276.1153.


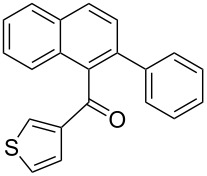


**[2-(Phenyl)naphthalen-1-yl](thiophen-3-yl)-methanone** (**3d**). Following the general MACOS benzannulation protocol, 2-(2-(thiophen-3-yl)-ethynyl)-benzaldehyde (**1d**) and phenylacetylene were reacted and 650 μL of the crude reaction mixture were collected. Purification by flash chromatography (15% ethyl acetate in hexane) afforded 63.7 mg of **3d** as a pale-yellow oil (62% yield). ^1^H NMR (400 MHz, CDCl_3_): δ 8.04 (d, *J* = 8.1 Hz, 1H), 7.96 (d, *J* = 8.1 Hz, 1H), 7.84 (d, *J* = 8.1 Hz, 1H), 7.61 (d, *J* = 8.1 Hz, 1H), 7.48–7.58 (m, 3H), 7.38–7.47 (m, 3H), 7.21–7.34 (m, 3H), 7.12–7.15 (m, 1H). ^13^C NMR (100 MHz, CDCl_3_): δ 198.6, 143.5, 140.3, 137.1, 136.4, 135.3, 132.4, 130.4, 129.6, 129.4, 128.3, 128.1, 127.6, 127.5, 127.3, 127.2, 126.4, 126.2, 125.5. Anal. Calcd. for C_21_H_14_OS: C, 80.22; H, 4.49; found C, 79.79; H, 4.13. HRMS calcd. for C_21_H_14_OS: 314.0765; found 314.0759.


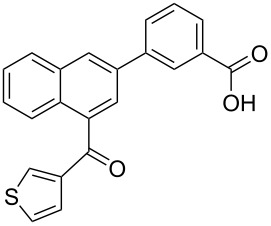


**3-[4-(Thiophene-3-carbonyl)naphthalen-2-yl]-benzoic acid** (**4e**). Following the general MACOS benzannulation protocol, 2-(2-(thiophen-3-yl)-ethynyl)-benzaldehyde (**1d**) and 3-ethynylbenzoic acid were reacted and 700 μL of the crude reaction mixture were collected. Purification by flash chromatography (10% methanol in dichloromethane) afforded 75.0 mg of **4e** as a yellow oil (60% yield). ^1^H NMR (400 MHz, CDCl_3_): δ 8.17 (s, 1H), 8.10 (d, *J* = 8.1 Hz, 1H), 7.93–8.00 (m, 2H), 7.82 (d, *J* = 8.1 Hz, 1H), 7.67 (d, *J* = 8.1 Hz, 1H), 7.48–7.63 (m, 4H), 7.33–7.43 (m, 2H), 7.15 (s, 1H). ^13^C NMR (150 MHz, CDCl_3_): δ 192.7, 171.3, 143.4, 140.6, 136.7, 135.7, 135.3, 134.6, 132.6, 130.8, 130.3, 129.7, 129.4, 129.1, 128.4, 128.1, 127.3, 127.2, 127.0, 126.6, 126.4, 125.5. HRMS calcd. for C_22_H_14_O_3_S: 358.0664; found 358.0666.


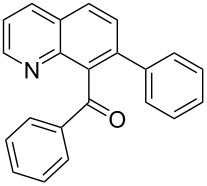


**Phenyl-(7-phenylquinolin-8-yl)-methanone** (**3f**). Following the general MACOS benzannulation protocol, 2-(2-phenylethynyl)-nicotinaldehyde (**1f**) and phenyl acetylene were reacted and 780 μL of the crude reaction mixture were collected. Purification by flash chromatography (20% ethyl acetate in pentane) afforded 77.8 mg of **3f** and **4f** isomers as pale-yellow oils (64% combined yield). (**3f**) ^1^H NMR (400 MHz, CDCl_3_): δ 8.88 (dd, *J* = 4.1, 1.5 Hz, 1H), 8.25 (d, *J* = 8.1 Hz, 1H), 8.01 (d, *J* = 8.1 Hz, 1H), 7.66–7.73 (m, 3H), 7.40–7.47 (m, 4H), 7.20–7.33 (m, 5H). ^13^C NMR (100 MHz, CDCl_3_): δ 198.8, 151.1, 146.6, 140.5, 139.3, 137.9, 137.4, 135.7, 133.0, 129.6, 129.3, 128.7, 128.6, 128.3, 128.2, 127.7, 127.0, 121.4. HRMS calcd. for C_22_H_15_NO: 309.1154; found 309.1145.


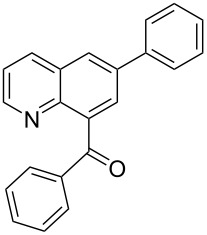


**Phenyl-(6-phenylquinolin-8-yl)-methanone** (**4f**). ^1^H NMR (400 MHz, CDCl_3_): δ 8.86 (dd, *J* = 4.1, 1.5 Hz, 1H), 8.29 (dd, *J* = 8.1, 1.5 Hz, 1H), 8.16 (d, *J* = 2.0 Hz, 1H), 8.03 (d, *J* = 2.0 Hz, 1H), 7.92 (dd, *J* = 8.1, 1.5 Hz, 2H), 7.75 (dd, *J* = 8.1, 1.5 Hz, 2H), 7.59 (t, *J* = 7.1 Hz, 1H), 7.49–7.56 (m, 2H), 7.40–7.49 (m, 4H). ^13^C NMR (100 MHz, CDCl_3_): δ 197.7, 150.8, 145.6, 139.8, 139.5, 138.7, 137.7, 136.2, 133.3, 130.3, 129.1, 128.5, 128.3, 128.1, 127.9, 127.5, 127.1, 122.0. Anal. Calcd. for C_22_H_15_NO: C, 85.40; N, 4.53; H, 4.89; found C, 84.98; N, 4.32; H, 4.62. HRMS calcd. for C_22_H_15_NO: 309.1154; found 309.1143.


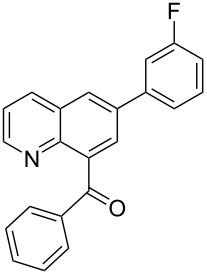


**[6-(3-Fluorophenyl)quinolin-8-yl](phenyl)methanone** (**4g**). Following the general MACOS benzannulation protocol, 2-(2-phenylethynyl)-nicotinaldehyde (**1f**) and 1-ethynyl-3-fluorobenzene were reacted and 750 μL of the crude reaction mixture were collected. Purification by flash chromatography (25% ethyl acetate in pentane) afforded 63.8 mg of **4g** as a colourless oil (54% yield). ^1^H NMR (400 MHz, CDCl_3_): δ 8.87 (dd, *J* = 4.0, 1.5 Hz, 1H), 8.29 (dd, *J* = 8.1, 1.5 Hz, 1H), 8.15 (d, *J* = 2.0 Hz, 1H), 7.99 (d, *J* = 2.0 Hz, 1H), 7.90 (dd, *J* = 8.1, 1.5 Hz, 2H), 7.60 (t, *J* = 7.1 Hz, 1H), 7.40–7.56 (m, 6H), 7.10–7.17 (m, 1H). ^13^C NMR (100 MHz, CDCl_3_): δ 197.6, 163.3 (^1^*J*^13^C-^19^F = 245.0 Hz), 151.1, 145.7, 141.8, 141.7, 140.1, 137.6, 137.5, 136.2, 133.4, 130.6 (^3^*J*^13^C-^19^F = 8.5 Hz), 130.2, 128.4, 127.5, 127.3, 123.1, 122.1, 114.9 (^2^*J*^13^C-^19^F = 22.0 Hz), 114.4 (^2^*J*^13^C-^19^F = 23.0 Hz). Anal. Calcd. for C_22_H_14_FNO: C, 80.72; N, 4.28; H, 4.31; found C, 80.77; N, 4.56; H, 4.09. HRMS calcd. for C_22_H_14_FNO: 327.1059; found 327.1046.


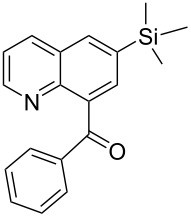


**Phenyl-[6-(trimethylsilyl)quinolin-8-yl]methanone** (**4h**). Following the general MACOS benzannulation protocol, 2-(2-phenylethynyl)-nicotinaldehyde (**1f**) and TMS acetylene were reacted and 800 μL of the crude reaction mixture were collected. Purification by flash chromatography (20% ethyl acetate in pentane) afforded 70.0 mg of **4h** as a pale-brown oil (58% yield). ^1^H NMR (400 MHz, CDCl_3_): δ 8.82 (dd, *J* = 4.1, 1.5 Hz, 1H), 8.21 (dd, *J* = 8.1, 1.5 Hz, 1H), 8.11 (d, *J* = 1.6 Hz, 1H), 7.84–7.87 (m, 3H), 7.55 (t, *J* = 7.6 Hz, 1H), 7.38–7.44 (m, 3H), 0.38 (s, 9H). ^13^C NMR (100 MHz, CDCl_3_): δ 198.5, 151.0, 146.4, 138.9, 138.3, 137.9, 136.0, 135.5, 133.2, 132.1, 130.2, 128.3, 127.5, 121.7, 0.87. Anal. Calcd. for C_19_H_19_NOSi: C, 74.71; N, 4.59; H, 6.27; found C, 74.93; N, 4.62; H, 6.07. HRMS calcd. for C_19_H_19_NOSi: 305.1236; found 305.1232.


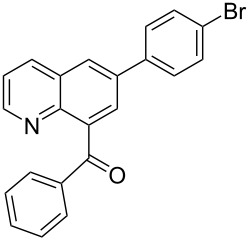


**[6-(4-Bromophenyl)quinolin-8-yl]-phenylmethanone** (**4i**). Following the general MACOS benzannulation protocol, 2-(2-phenylethynyl)-nicotinaldehyde (**1f**) and 1-bromo-4-ethynylbenzene were reacted and 700 μL of the crude reaction mixture were collected. Purification by flash chromatography (20% ethyl acetate in pentane) afforded 70.0 mg of **4i** as a colourless oil (52% yield). ^1^H NMR (400 MHz, CD_2_Cl_2_): δ 8.83 (dd, *J* = 4.1, 1.5 Hz, 1H), 8.35 (dd, *J* = 8.1, 1.5 Hz, 1H), 8.21 (d, *J* = 2.0 Hz, 1H), 8.00 (d, *J* = 2.0 Hz, 1H), 7.85 (dd, *J* = 8.1, 1.5 Hz, 2H), 7.60–7.76 (m, 4H), 7.44–7.56 (m, 3H), 7.34 (q, *J* = 9.0 Hz, 1H). ^13^C NMR (150 MHz, CD_2_Cl_2_): δ 197.3, 150.7, 145.4, 140.0, 138.5, 137.8, 137.5, 136.2, 133.2, 132.1, 129.8, 128.9, 128.4, 128.3, 127.2, 126.9, 122.3, 122.1. HRMS calcd. for C_22_H_14_BrNO [M+H]^+^: 388.0337; found 388.0337.


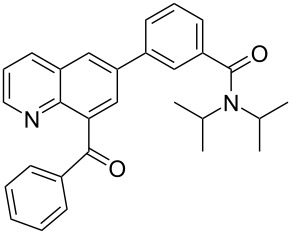


**3-(8-benzoylquinolin-6-yl)-*N,N*-diisopropylbenzamide** (**4j**). Following the general MACOS benzannulation protocol, 2-(2-phenylethynyl)-nicotinaldehyde (**1f**) and 3-ethynyl-N,N-diisopropylbenzamide were reacted and 840 μL of the crude reaction mixture were collected. Purification by flash chromatography (30% ethyl acetate in pentane) afforded 72.0 mg of **4j** as a colourless oil (40% yield). ^1^H NMR (600 MHz, CD_2_Cl_2_): δ 8.81 (dd, *J* = 3.8, 1.6 Hz, 1H), 8.35 (dd, *J* = 8.1, 1.6 Hz, 1H), 8.25 (d, *J* = 2.1 Hz, 1H), 8.03 (d, *J* = 2.1 Hz, 1H), 7.84 (dd, *J* = 8.1, 1.6 Hz, 2H), 7.79 (d, *J* = 8.1 Hz, 1H), 7.70 (s, 1H), 7.61 (t, *J* = 7.6 Hz, 1H), 7.56 (t, *J* = 7.6 Hz, 1H), 7.44–7.52 (m, 3H), 7.35 (d, *J* = 8.1 Hz, 1H), 3.90 (br s, 1H), 3.54 (br s, 1H), 1.55 (s, 6H), 1.16 (s, 6H). ^13^C NMR (150 MHz, CD_2_Cl_2_ 10 °C): δ 197.6, 170.2, 150.7, 145.5, 140.2, 140.0, 139.9, 138.1, 137.8, 136.3, 133.3, 129.9, 129.2, 128.5, 128.4, 127.5, 127.4, 127.2, 124.9, 124.6, 122.2, 51.0, 45.7, 20.4. HRMS calcd. for C_29_H_28_N_2_O_2_: 436.2151; found 436.2147.

## Supporting Information

Spectra for compounds made in this manuscript are available as supporting information.

File 1 NMR spectra of compounds **1d**, **2j**, **3c**, **3d**, **3f**, **4c** and **4e**–**j**.
