# Supplementary material for: Gold film-catalysed benzannulation by Microwave-Assisted, Continuous Flow Organic Synthesis (MACOS)
Source: Beilstein J Org Chem. 2009 Jul 21;5:35. doi: 10.3762/bjoc.5.35 (PMC2748690; doi:10.3762/bjoc.5.35)
Supplement: File 1 — NMR spectra of compounds 1d, 2j, 3c, 3d, 3f, 4c and 4e–j. [file Beilstein_J_Org_Chem-05-35-s001.pdf]

Supporting Information

Gold film-catalysed benzannulation by Microwave-Assisted, Continuous Flow Organic Synthesis (MACOS)

Gjergji Shore, Michael Tsimerman and Michael G. Organ*

Department of Chemistry, York University, 4700 Keele Street, Toronto, Ontario, M3J 1P3, Canada
e-mail: organ@yorku.ca

**Contents**	**Page**
^{1}H NMR Spectra for compound **1d**	S3
^{13}C NMR Spectra for compound **1d**	S4
^{1}H NMR Spectra for compound **2j**	S5
^{13}C NMR Spectra for compound **2j**	S6
^{1}H NMR Spectra for compound **3c**	S7
^{13}C NMR Spectra for compound **3c**	S8
^{1}H NMR Spectra for compound **4c**	S9
^{13}C NMR Spectra for compound **4c**	S10
^{1}H NMR Spectra for compound **3d**	S11
^{13}C NMR Spectra for compound **3d**	S12
^{1}H NMR Spectra for compound **4e**	S13
^{13}C NMR Spectra for compound **4e**	S14
^{1}H NMR Spectra for compound **3f**	S15
^{13}C NMR Spectra for compound **3f**	S16
^{1}H NMR Spectra for compound **4f**	S17
^{13}C NMR Spectra for compound **4f**	S18
^{1}H NMR Spectra for compound **4g**	S19

^{13}C NMR Spectra for compound **4g** S20
^{1}H NMR Spectra for compound **4h** S21
^{13}C NMR Spectra for compound **4h** S22
^{1}H NMR Spectra for compound **4i** S23
^{13}C NMR Spectra for compound **4i** S24
^{1}H NMR Spectra for compound **4j** S25
^{13}C NMR Spectra for compound **4j** S26
References S27

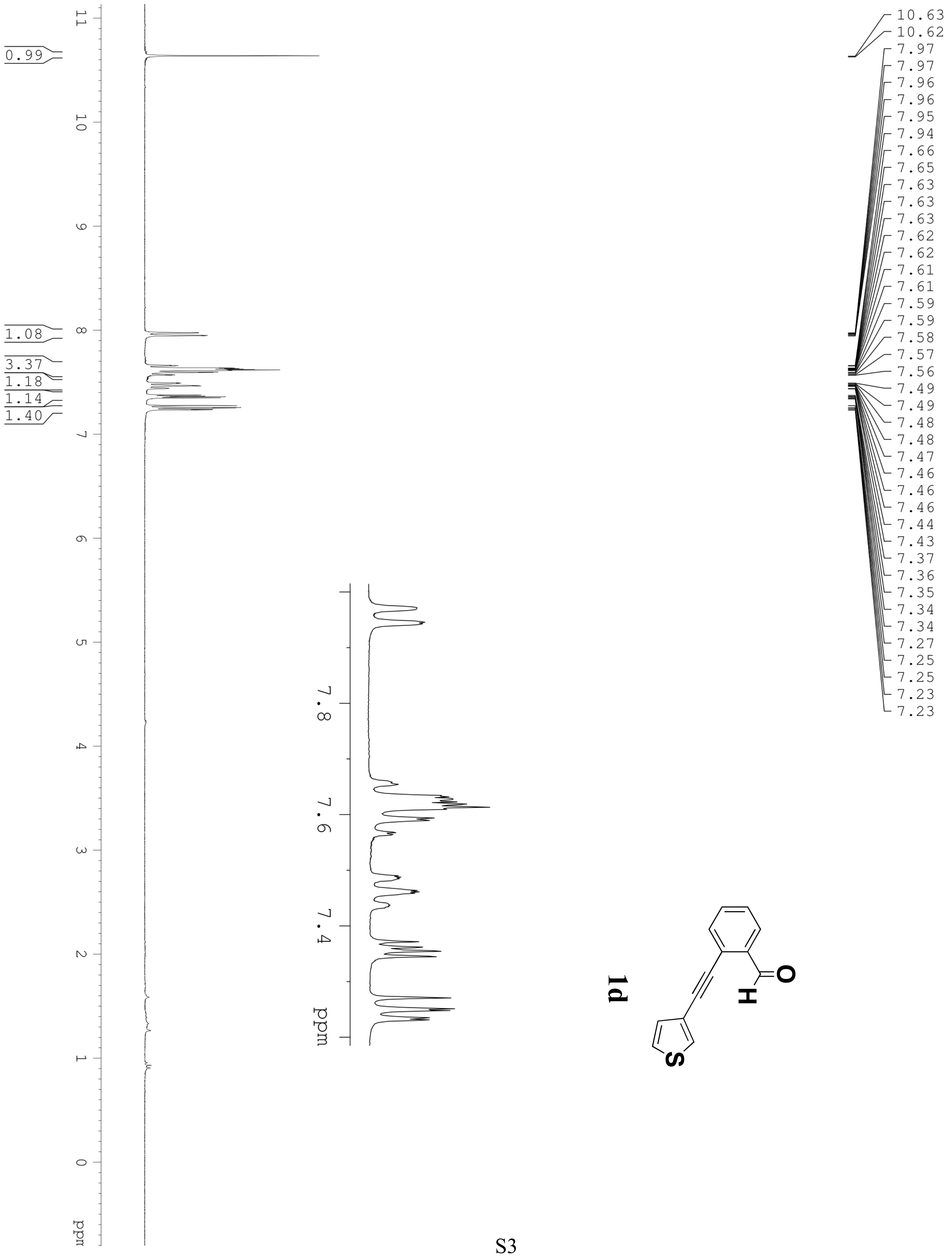
10.63
10.62
7.97
7.97
7.96
7.96
7.95
7.94
7.66
7.65
7.63
7.63
7.63
7.62
7.62
7.61
7.61
7.59
7.59
7.58
7.57
7.56
7.49
7.49
7.48
7.48
7.47
7.46
7.46
7.46
7.44
7.43
7.37
7.36
7.35
7.34
7.34
7.27
7.25
7.25
7.23
7.23
0.99
1.08
3.37
1.18
1.14
1.40
ppm
1d

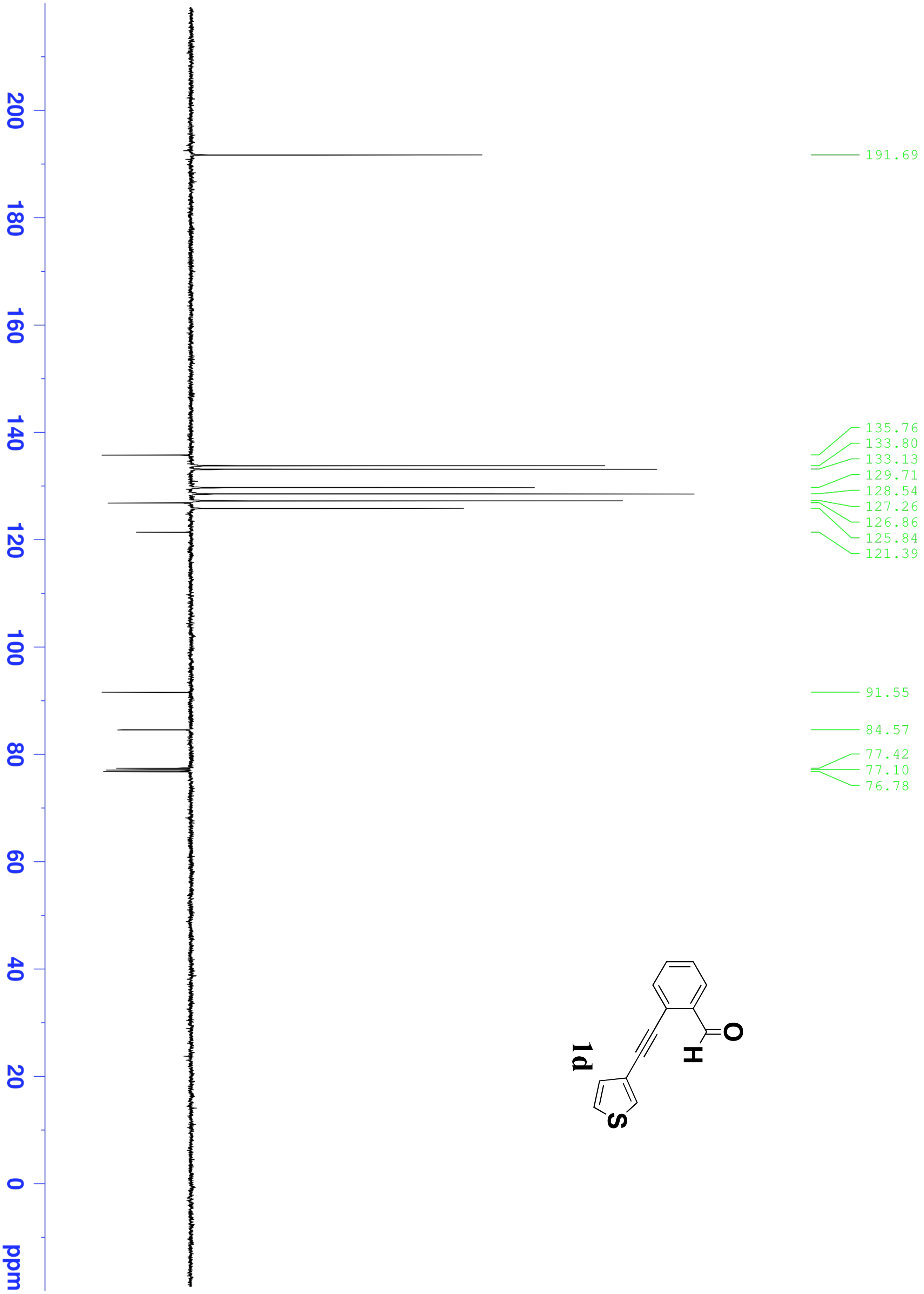
191.69
135.76
133.80
133.13
129.71
128.54
127.26
126.86
125.84
121.39
91.55
84.57
77.42
77.10
76.78
200
180
160
140
120
100
80
60
40
20
0
ppm
O
H
S
1d

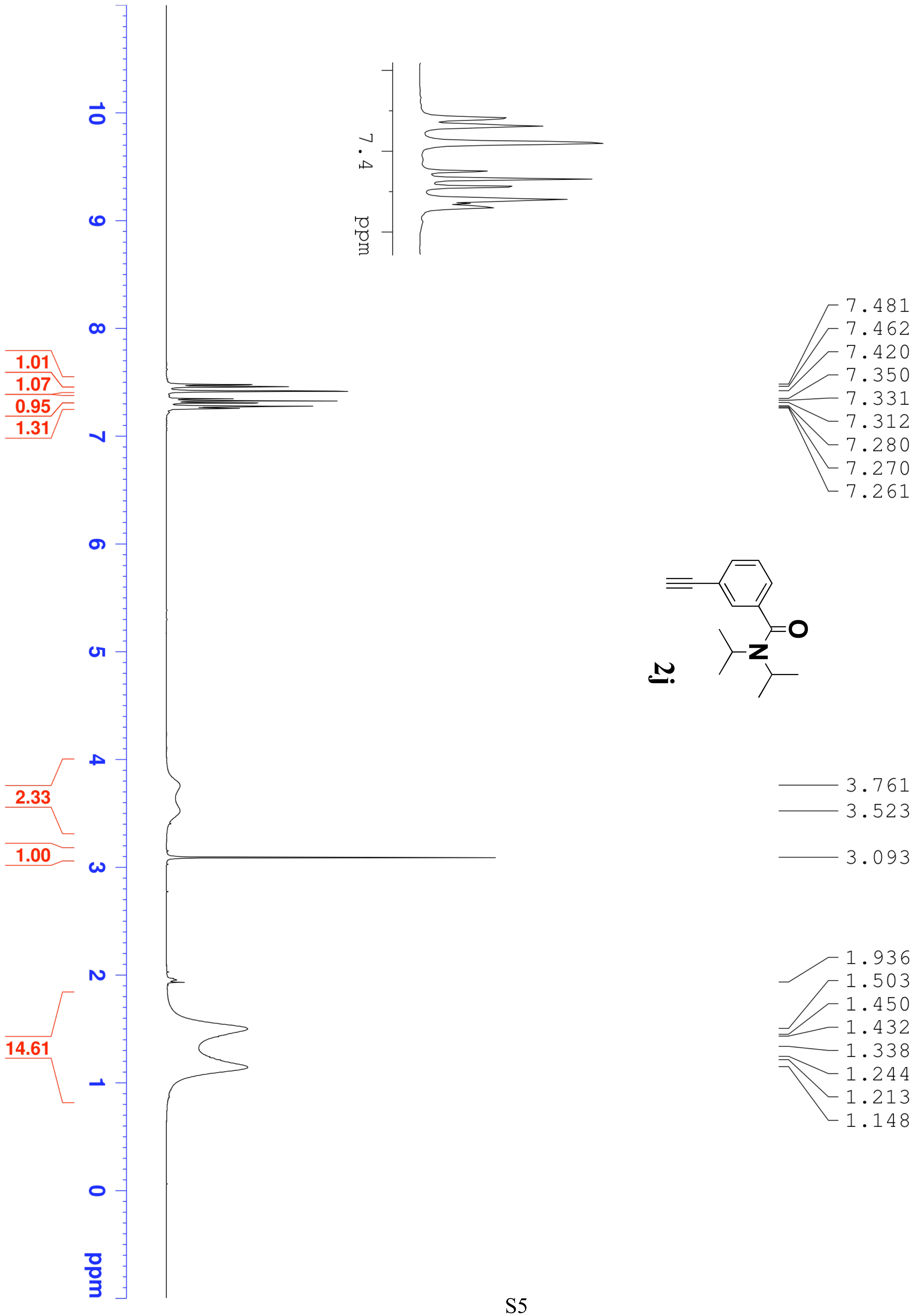

7.481
7.462
7.420
7.350
7.331
7.312
7.280
7.270
7.261
3.761
3.523
3.093
1.936
1.503
1.450
1.432
1.338
1.244
1.213
1.148
1.01
1.07
0.95
1.31
2.33
1.00
14.61
2j
ppm

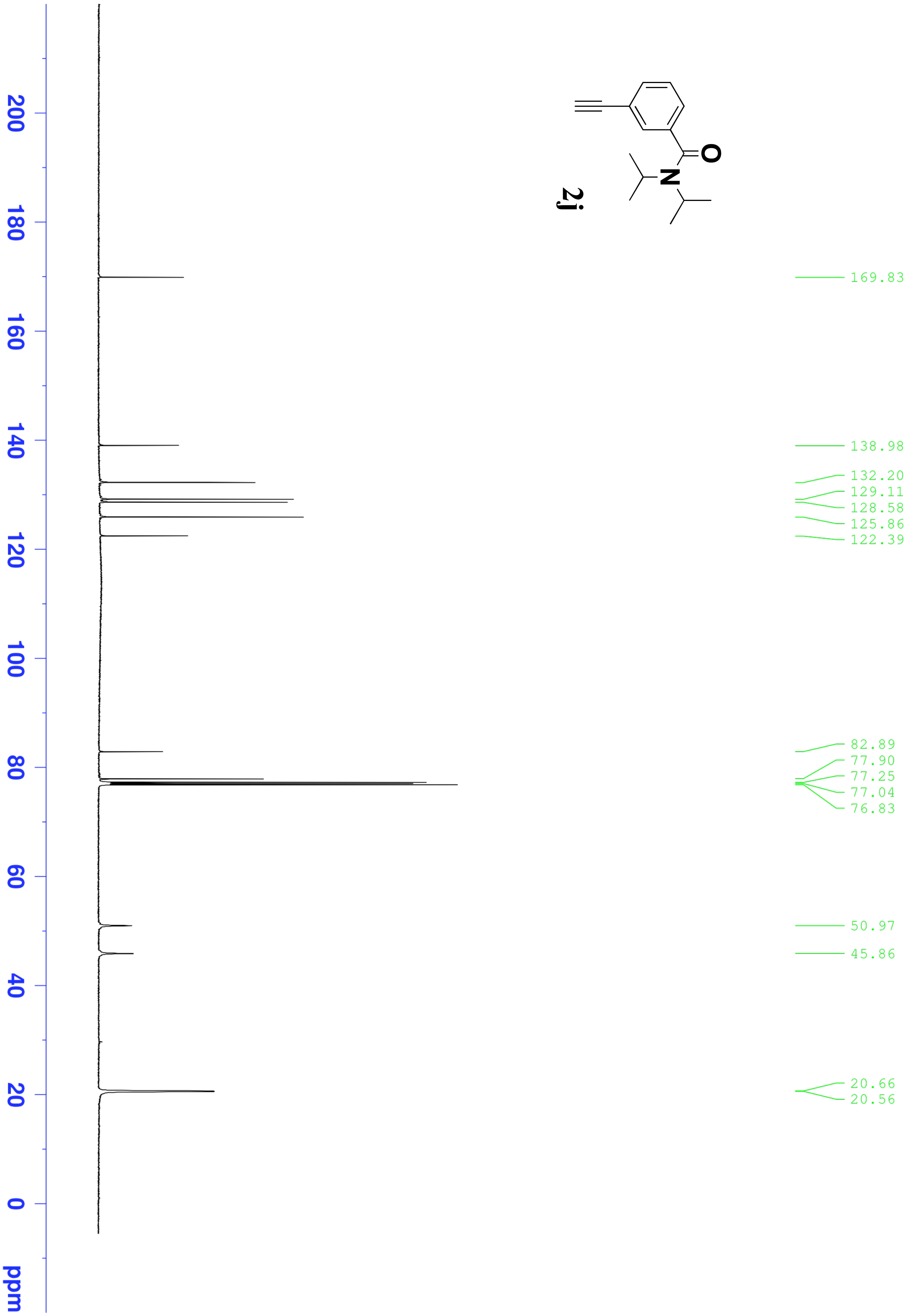

2j
169.83
138.98
132.20
129.11
128.58
125.86
122.39
82.89
77.90
77.25
77.04
76.83
50.97
45.86
20.66
20.56
200
180
160
140
120
100
80
60
40
20
0
ppm

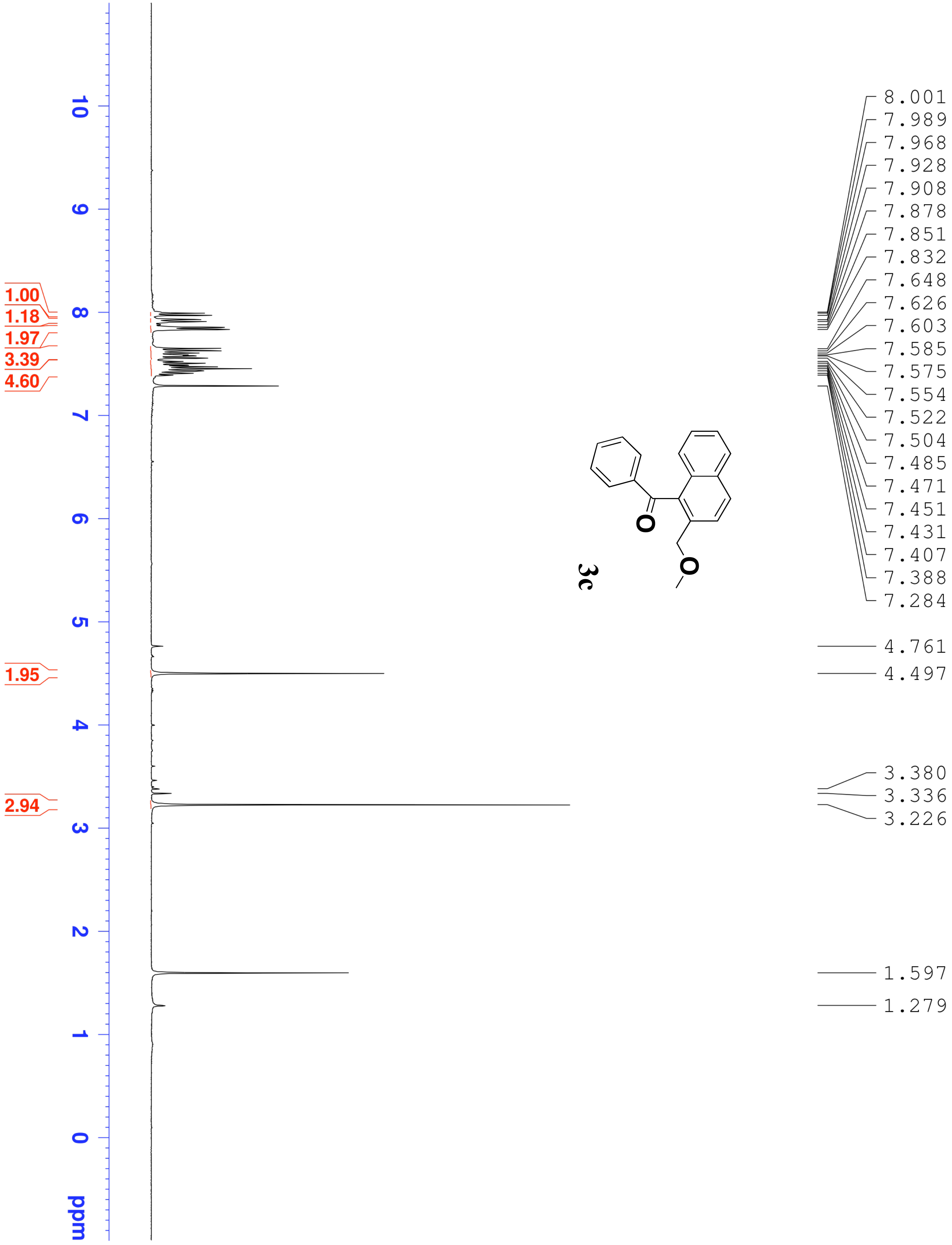

8.001
7.989
7.968
7.928
7.908
7.878
7.851
7.832
7.648
7.626
7.603
7.585
7.575
7.554
7.522
7.504
7.485
7.471
7.451
7.431
7.407
7.388
7.284
4.761
4.497
3.380
3.336
3.226
1.597
1.279
1.00
1.18
1.97
3.39
4.60
1.95
2.94
10
9
8
7
6
5
4
3
2
1
0
ppm
3c

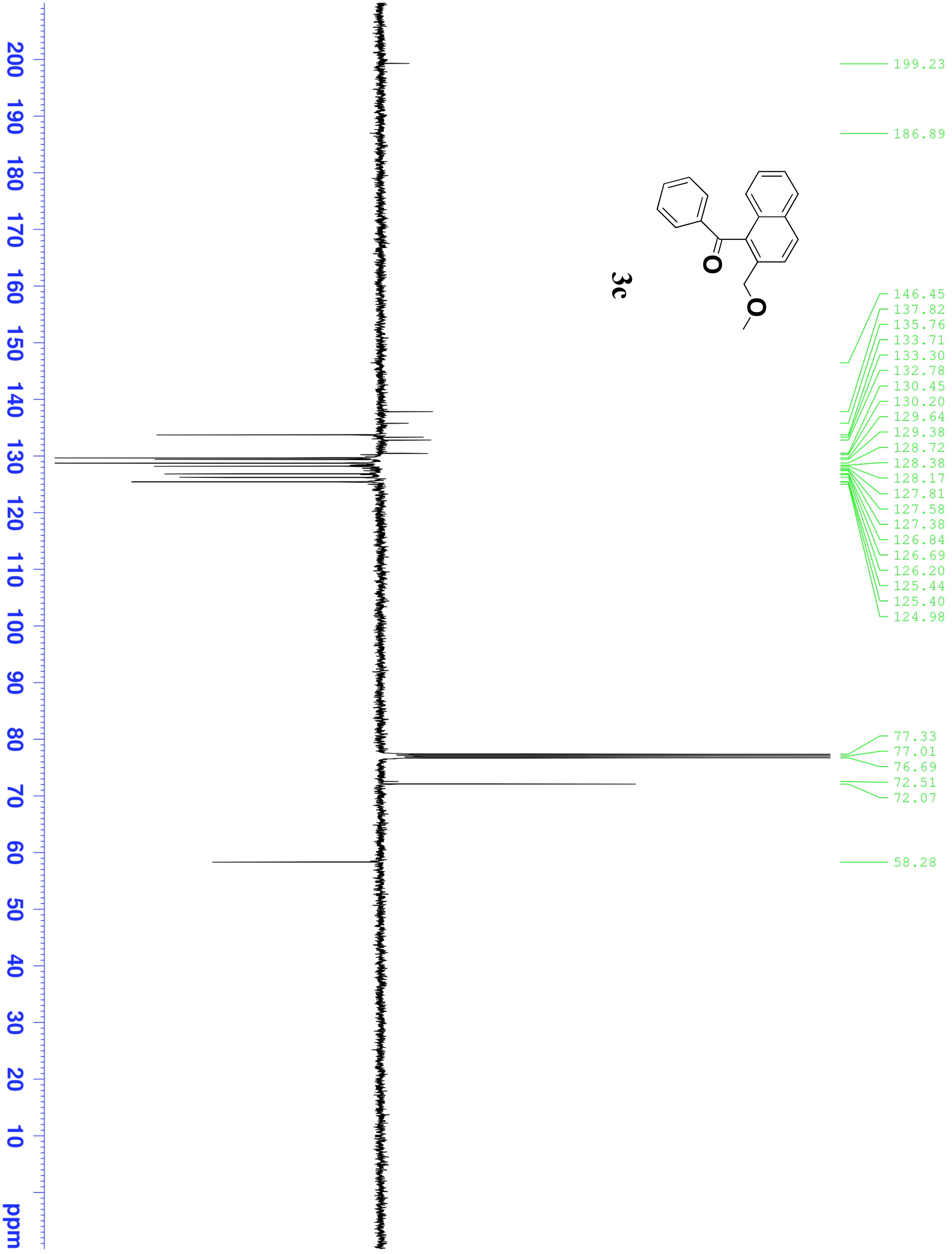
3c
199.23
186.89
146.45
137.82
135.76
133.71
133.30
132.78
130.45
130.20
129.64
129.38
128.72
128.38
128.17
127.81
127.58
127.38
126.84
126.69
126.20
125.44
125.40
124.98
77.33
77.01
76.69
72.51
72.07
58.28
200
190
180
170
160
150
140
130
120
110
100
90
80
70
60
50
40
30
20
10
ppm

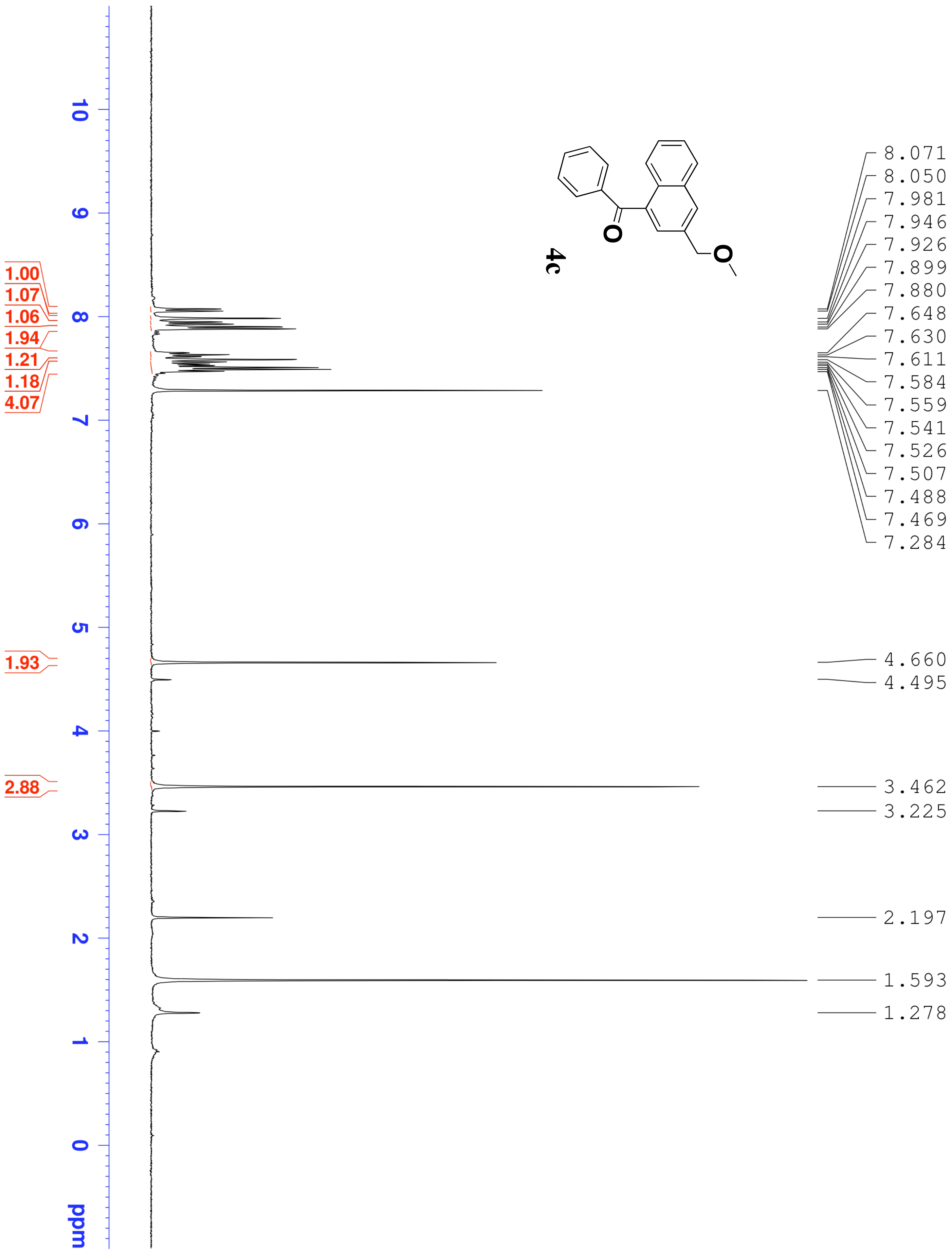
4c
8.071
8.050
7.981
7.946
7.926
7.899
7.880
7.648
7.630
7.611
7.584
7.559
7.541
7.526
7.507
7.488
7.469
7.284
4.660
4.495
3.462
3.225
2.197
1.593
1.278
1.00
1.07
1.06
1.94
1.21
1.18
4.07
1.93
2.88
10
9
8
7
6
5
4
3
2
1
0
ppm

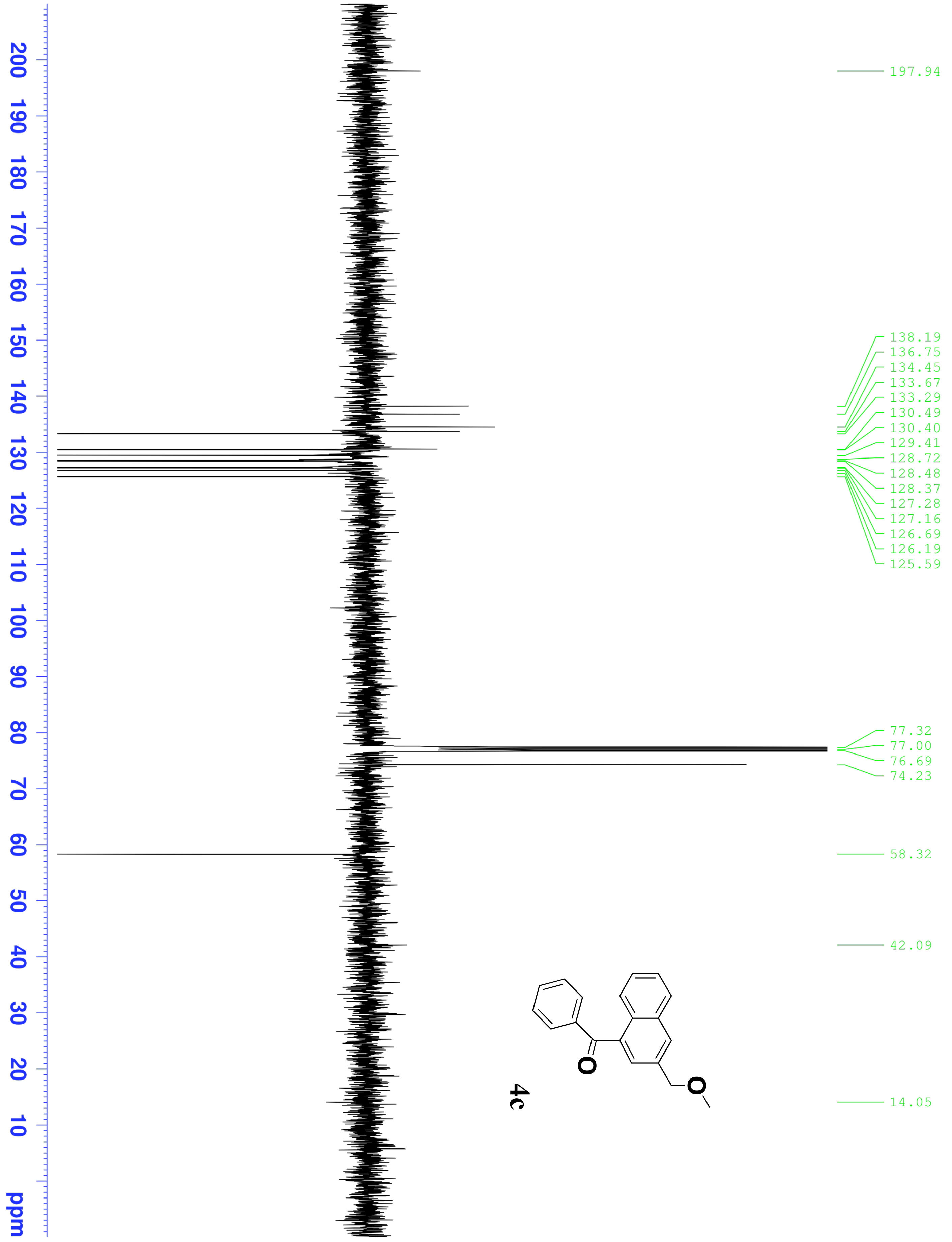
197.94
138.19
136.75
134.45
133.67
133.29
130.49
130.40
129.41
128.72
128.48
128.37
127.28
127.16
126.69
126.19
125.59
77.32
77.00
76.69
74.23
58.32
42.09
14.05
200
190
180
170
160
150
140
130
120
110
100
90
80
70
60
50
40
30
20
10
ppm
4c

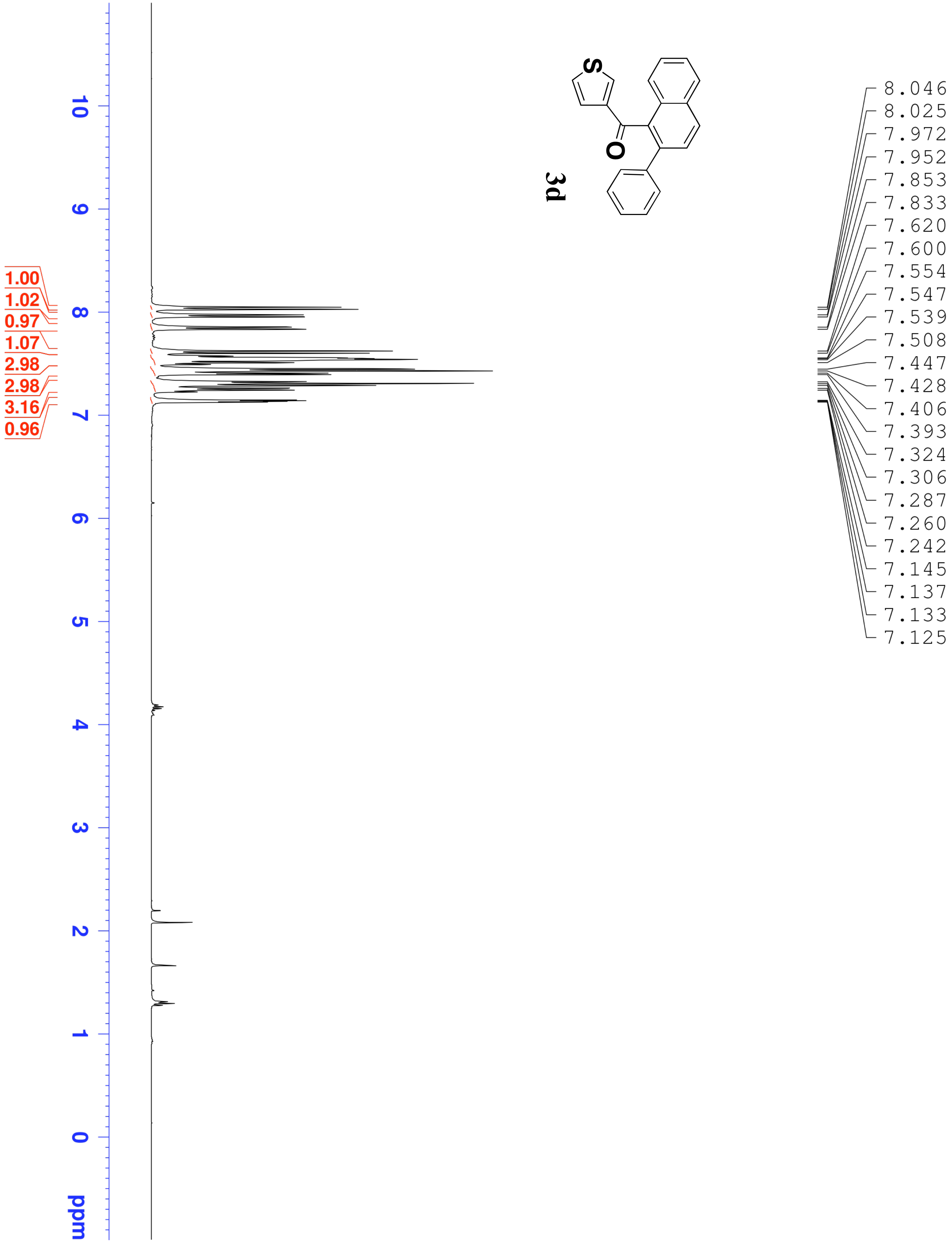
3d
S
O
8.046
8.025
7.972
7.952
7.853
7.833
7.620
7.600
7.554
7.547
7.539
7.508
7.447
7.428
7.406
7.393
7.324
7.306
7.287
7.260
7.242
7.145
7.137
7.133
7.125
1.00
1.02
0.97
1.07
2.98
2.98
3.16
0.96
10
9
8
7
6
5
4
3
2
1
0
ppm

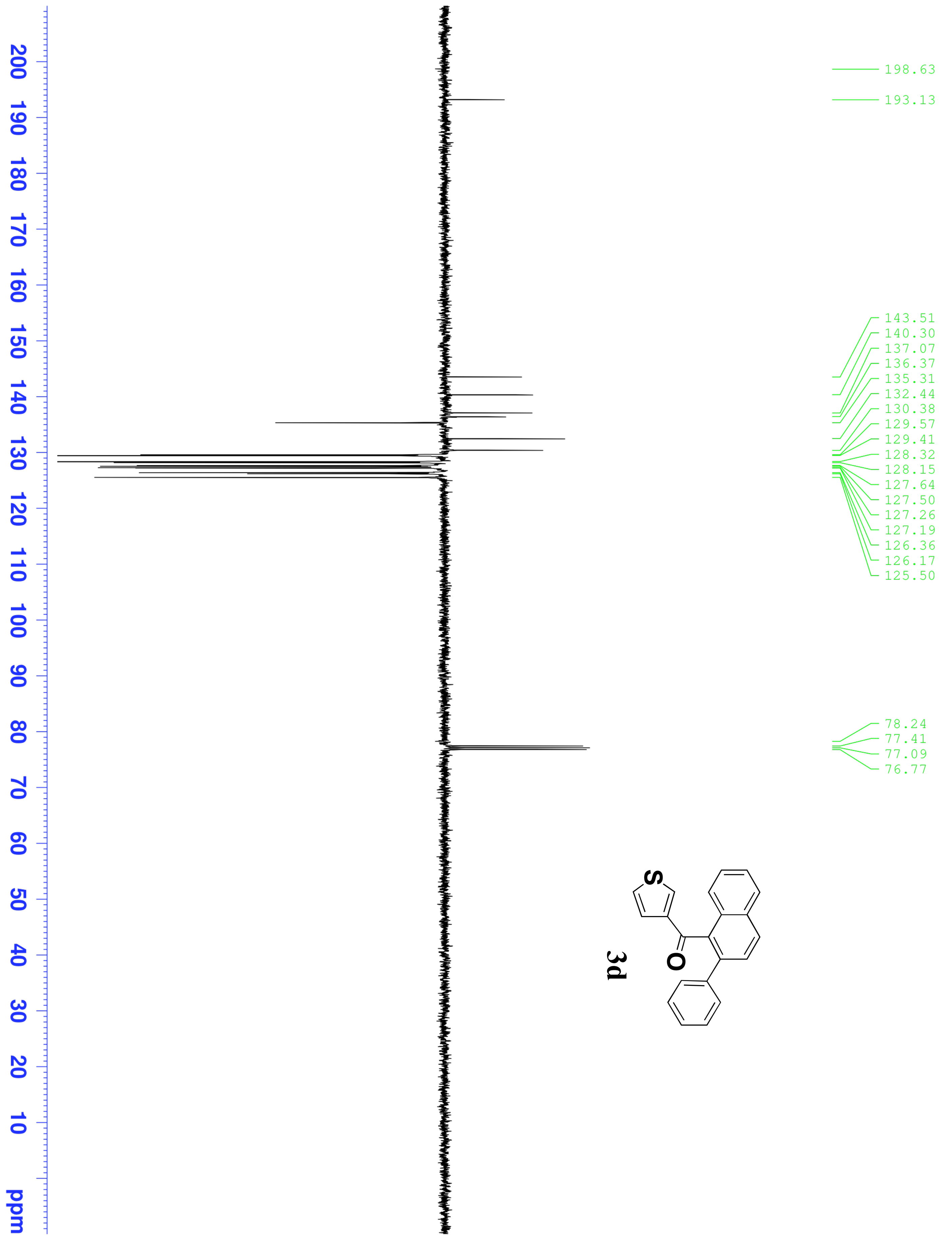
198.63
193.13
143.51
140.30
137.07
136.37
135.31
132.44
130.38
129.57
129.41
128.32
128.15
127.64
127.50
127.26
127.19
126.36
126.17
125.50
78.24
77.41
77.09
76.77
3d
ppm

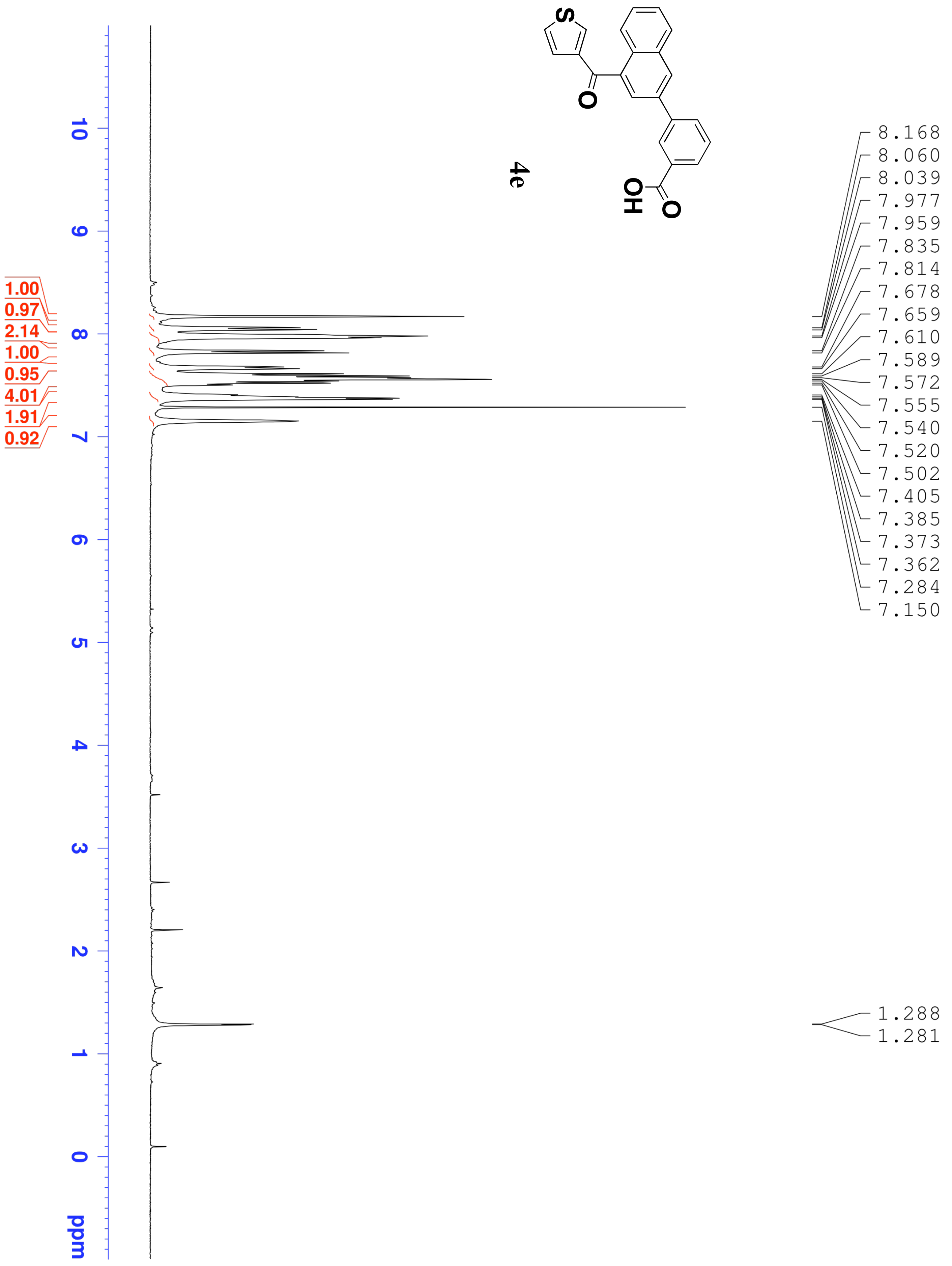
4e
OH
O
S
8.168
8.060
8.039
7.977
7.959
7.835
7.814
7.678
7.659
7.610
7.589
7.572
7.555
7.540
7.520
7.502
7.405
7.385
7.373
7.362
7.284
7.150
1.288
1.281
1.00
0.97
2.14
1.00
0.95
4.01
1.91
0.92
10
9
8
7
6
5
4
3
2
1
0
ppm

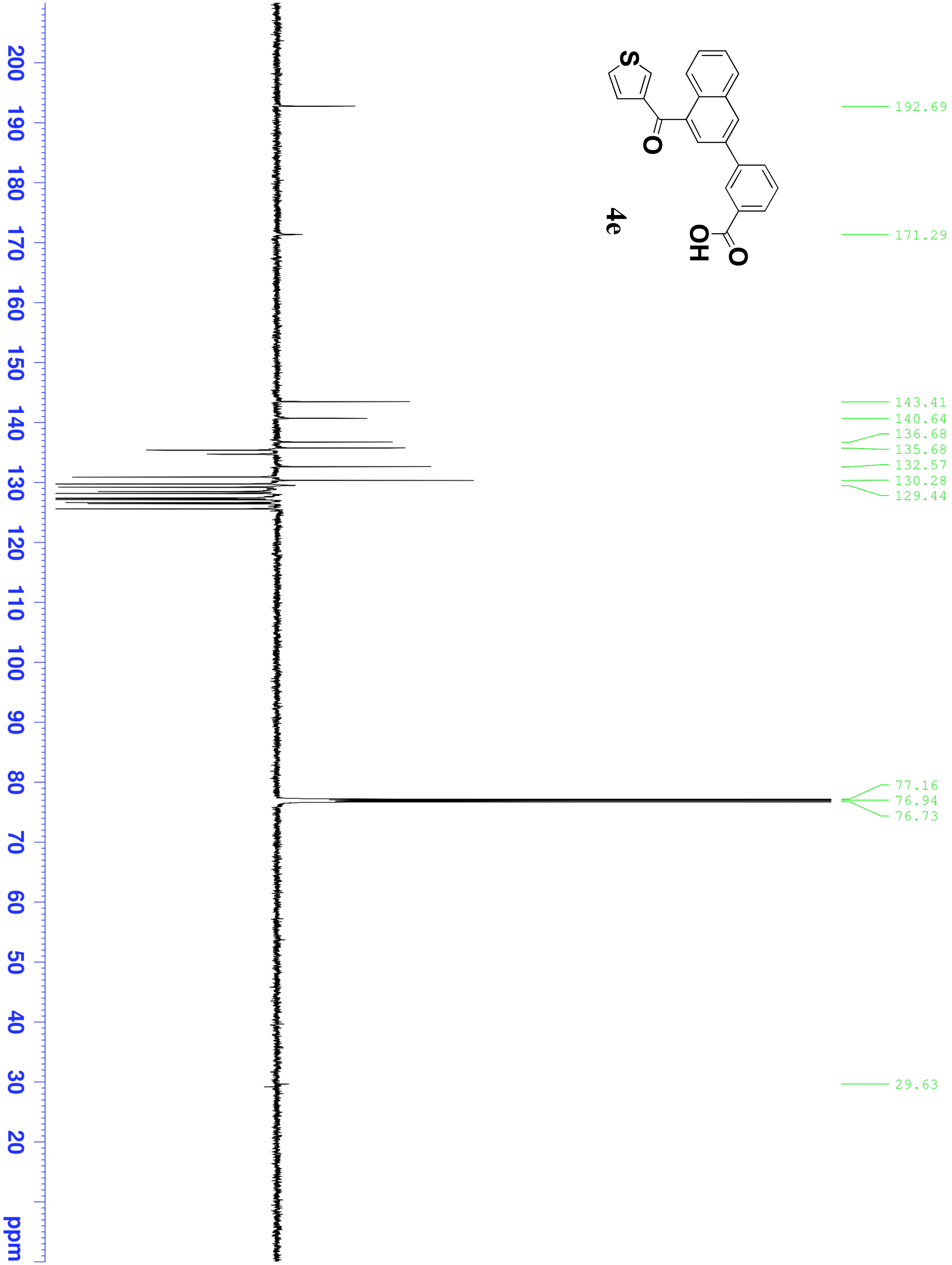

192.69
171.29
143.41
140.64
136.68
135.68
132.57
130.28
129.44
77.16
76.94
76.73
29.63
4e
200
190
180
170
160
150
140
130
120
110
100
90
80
70
60
50
40
30
20
ppm

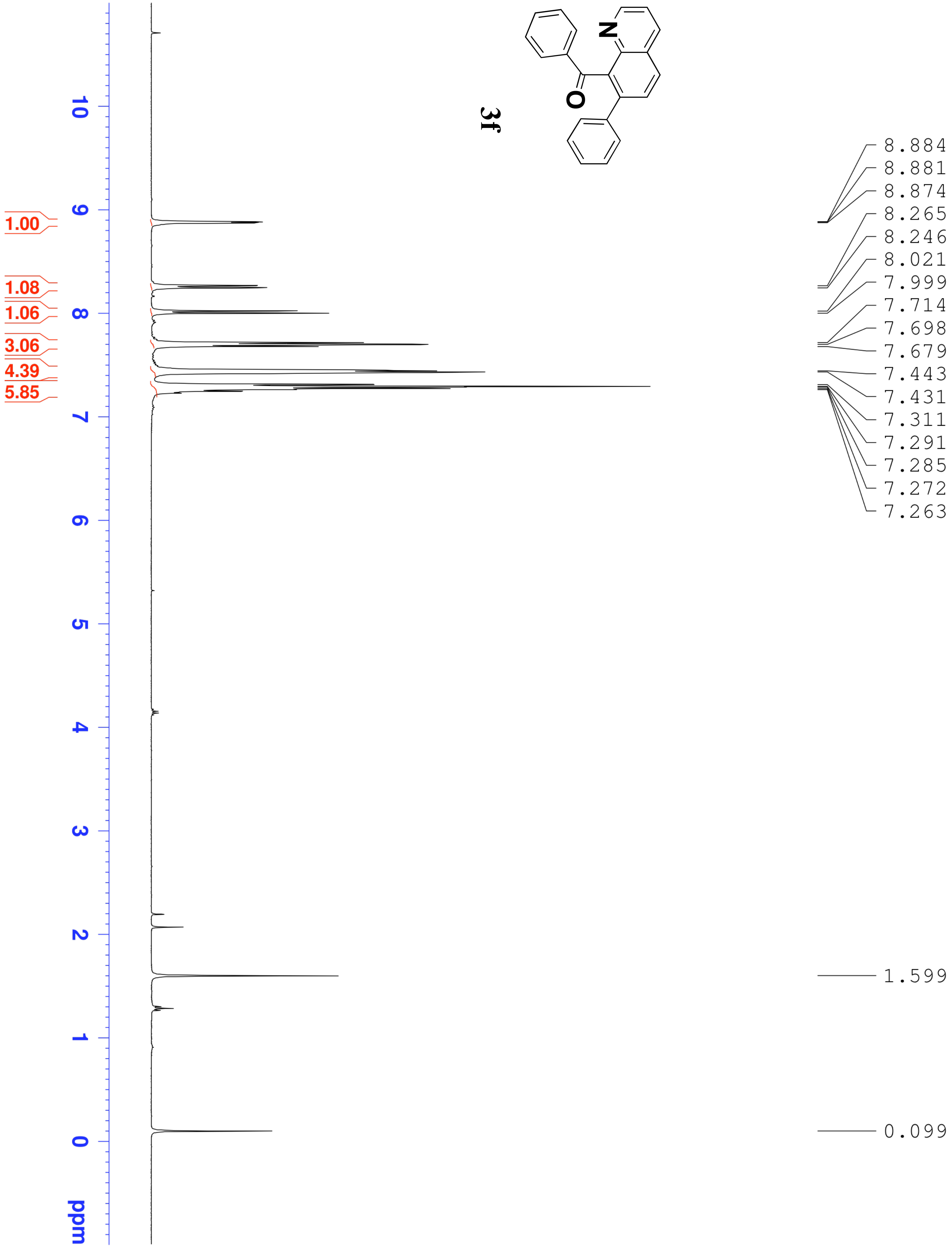
3f
N
O
8.884
8.881
8.874
8.265
8.246
8.021
7.999
7.714
7.698
7.679
7.443
7.431
7.311
7.291
7.285
7.272
7.263
1.599
0.099
1.00
1.08
1.06
3.06
4.39
5.85
10
9
8
7
6
5
4
3
2
1
0
ppm

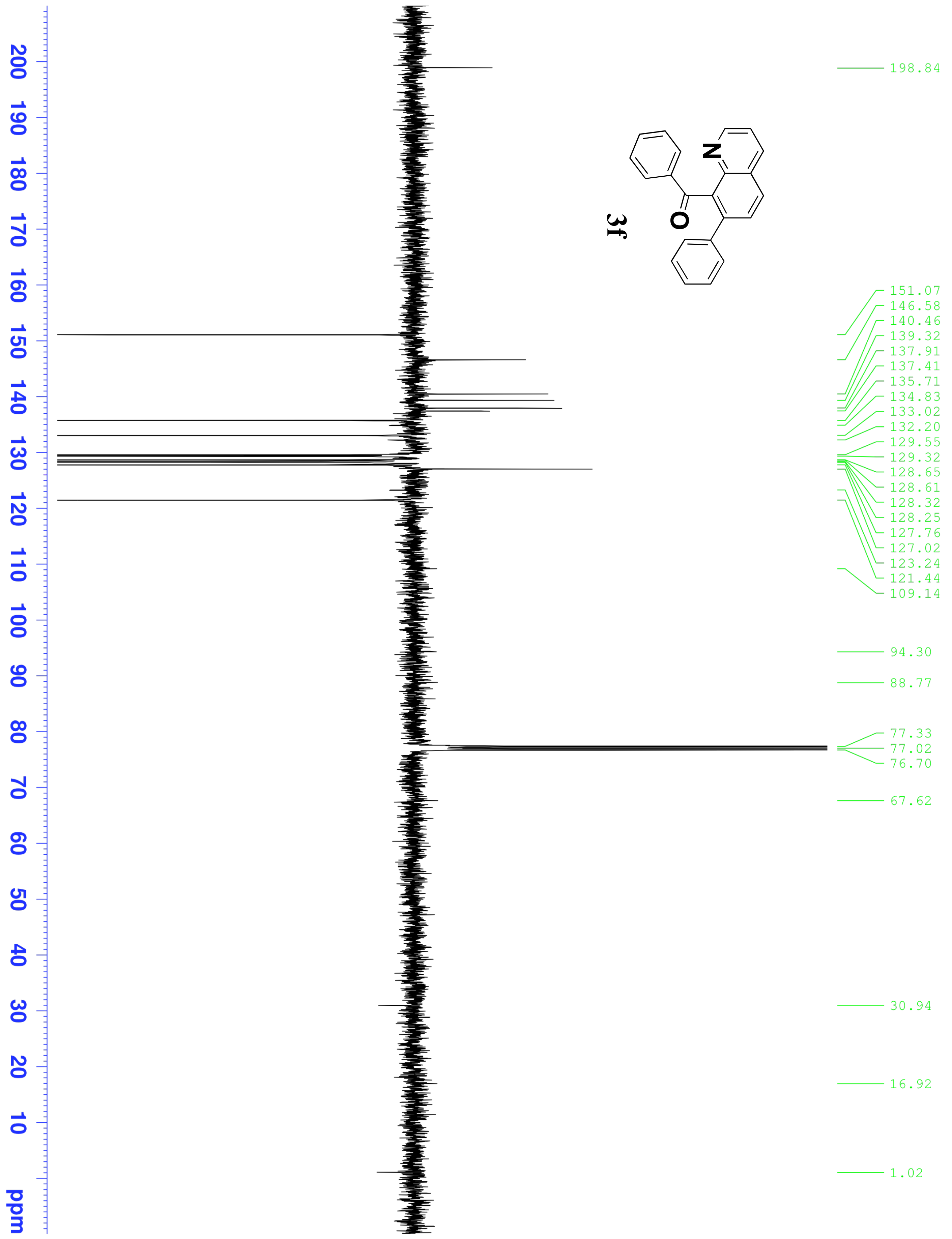
3f
N
O
198.84
151.07
146.58
140.46
139.32
137.91
137.41
135.71
134.83
133.02
132.20
129.55
129.32
128.65
128.61
128.32
128.25
127.76
127.02
123.24
121.44
109.14
94.30
88.77
77.33
77.02
76.70
67.62
30.94
16.92
1.02
200
190
180
170
160
150
140
130
120
110
100
90
80
70
60
50
40
30
20
10
ppm

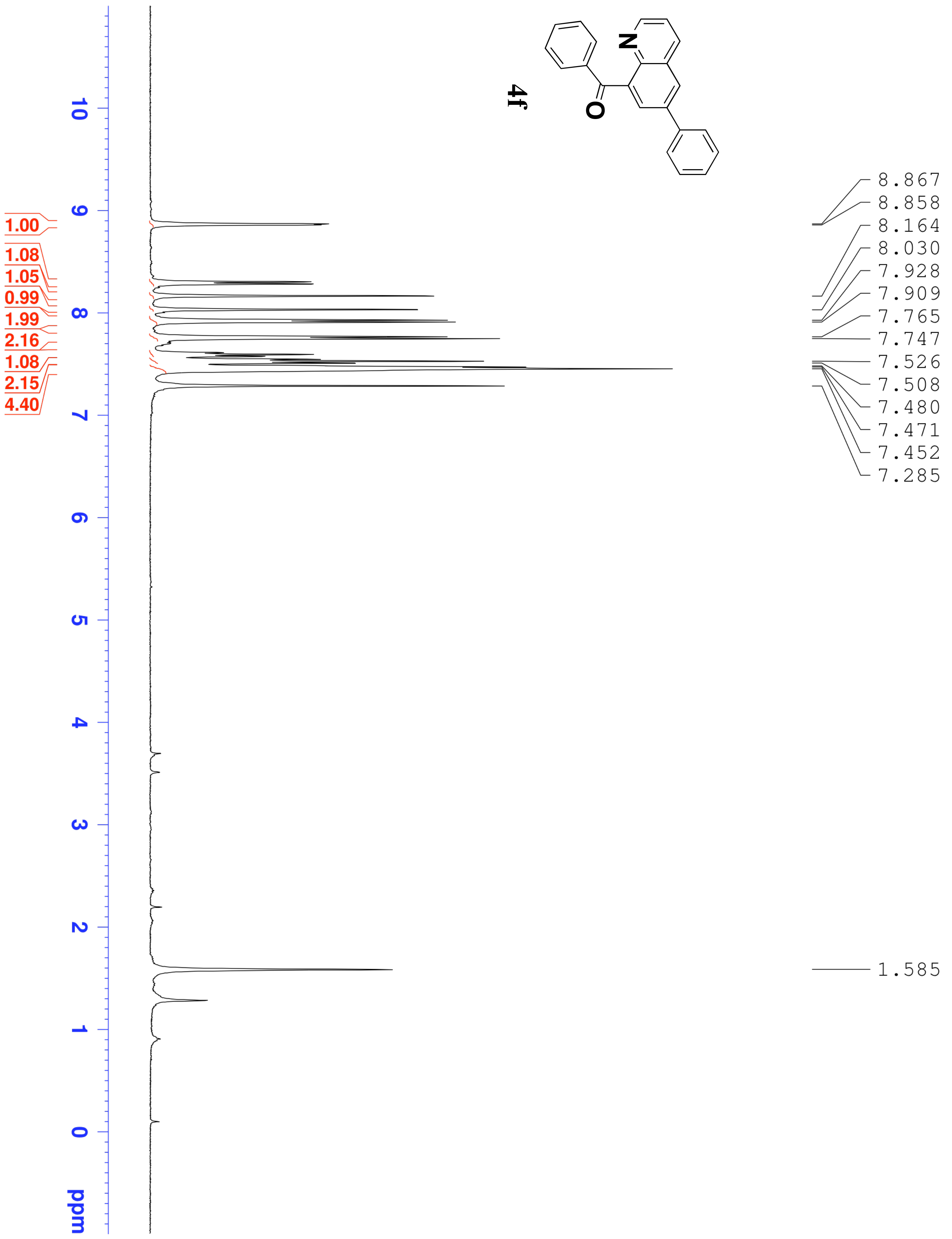
4f
N
O
8.867
8.858
8.164
8.030
7.928
7.909
7.765
7.747
7.526
7.508
7.480
7.471
7.452
7.285
1.585
1.00
1.08
1.05
0.99
1.99
2.16
1.08
2.15
4.40
10
9
8
7
6
5
4
3
2
1
0
ppm

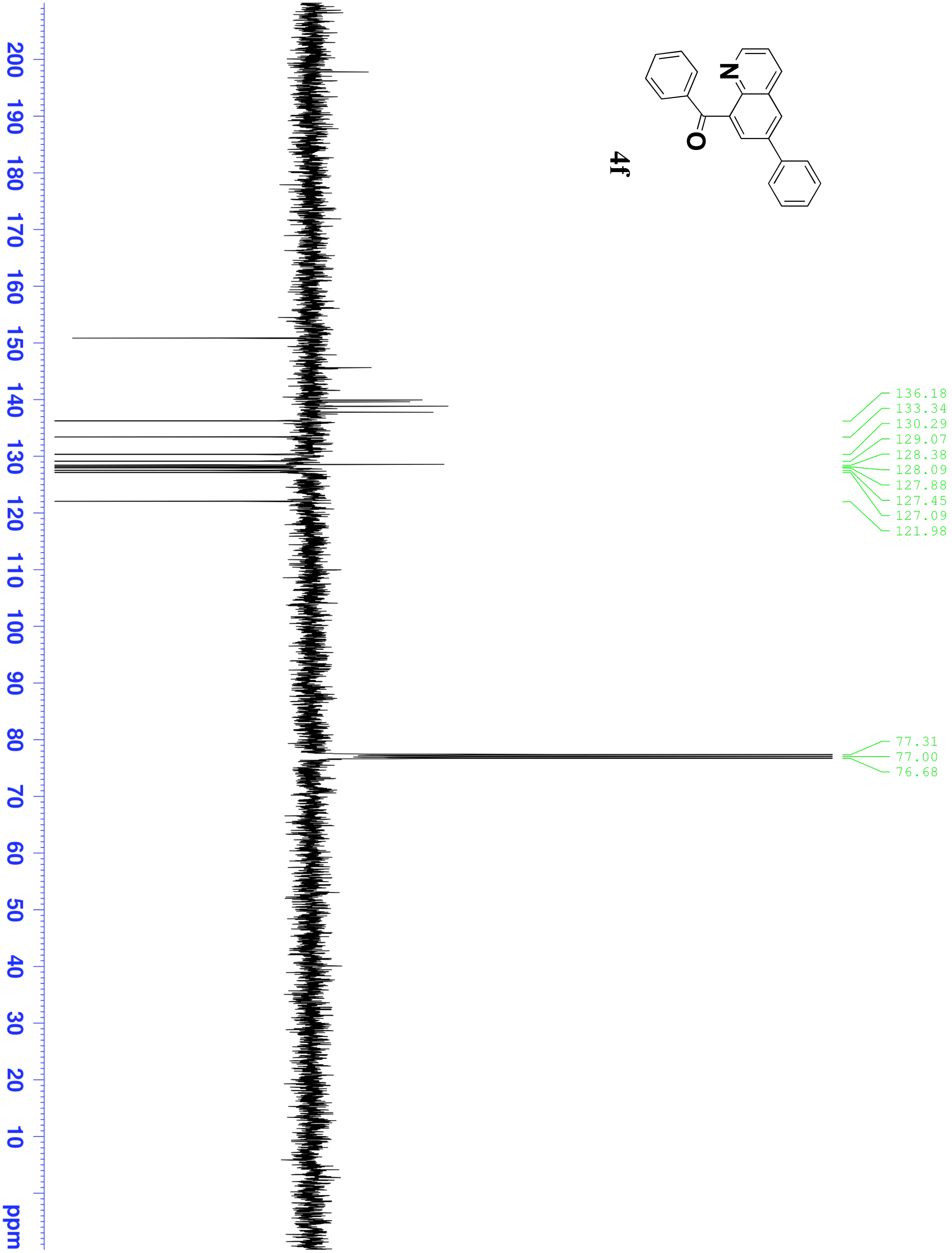

4f
136.18
133.34
130.29
129.07
128.38
128.09
127.88
127.45
127.09
121.98
77.31
77.00
76.68
200
190
180
170
160
150
140
130
120
110
100
90
80
70
60
50
40
30
20
10
ppm

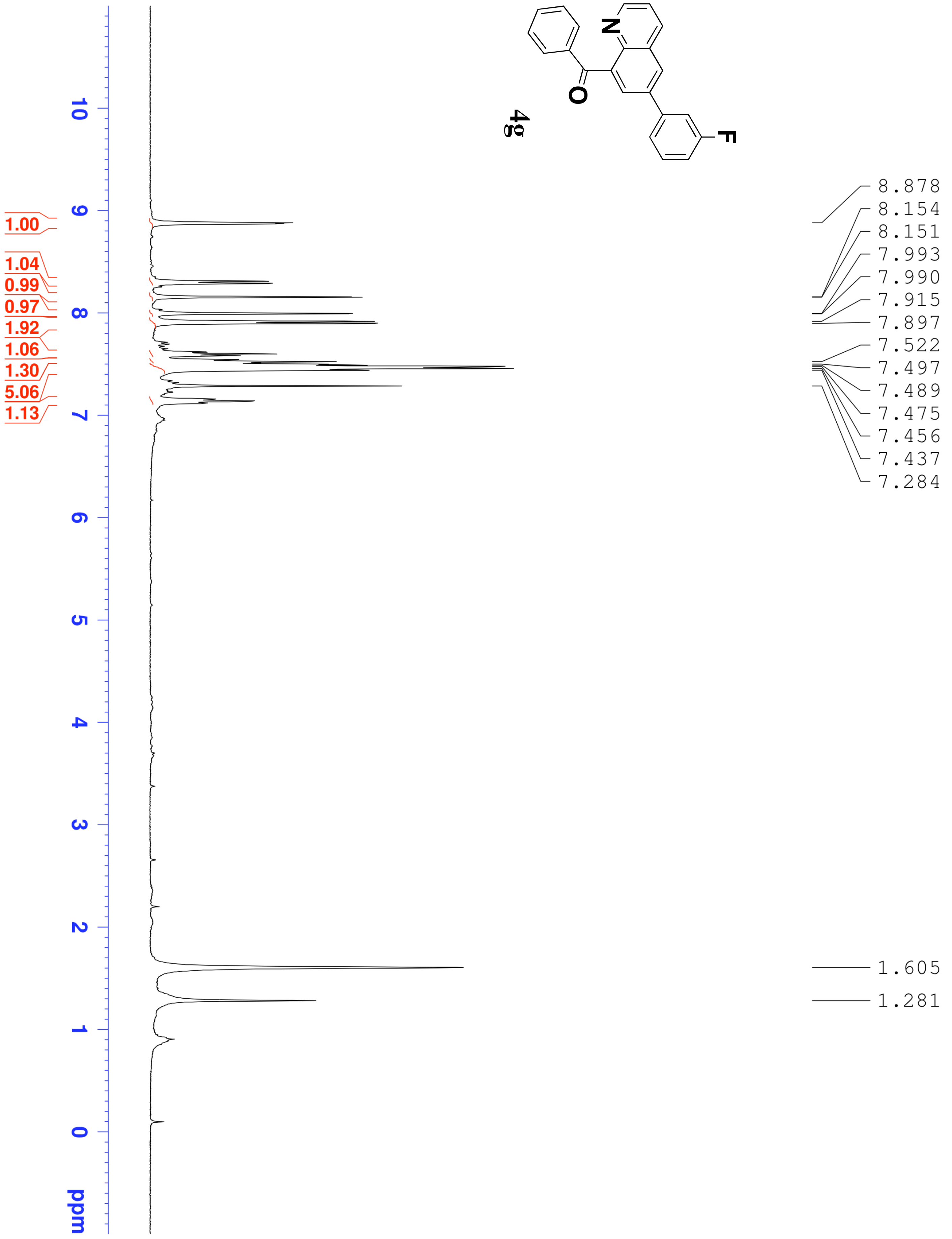
4g
8.878
8.154
8.151
7.993
7.990
7.915
7.897
7.522
7.497
7.489
7.475
7.456
7.437
7.284
1.605
1.281
1.00
1.04
0.99
0.97
1.92
1.06
1.30
5.06
1.13
10
9
8
7
6
5
4
3
2
1
0
ppm

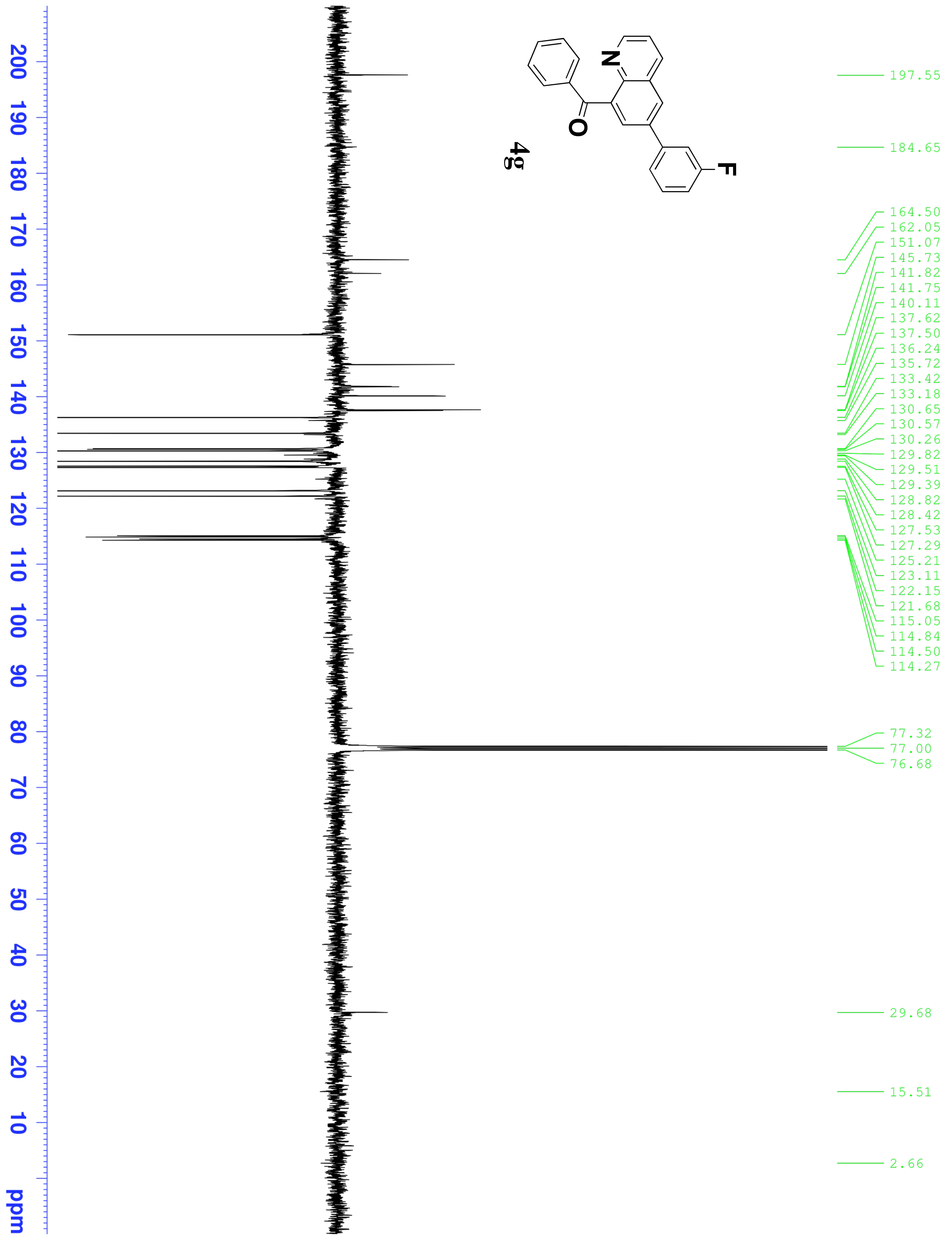
4g
N
O
F
197.55
184.65
164.50
162.05
151.07
145.73
141.82
141.75
140.11
137.62
137.50
136.24
135.72
133.42
133.18
130.65
130.57
130.26
129.82
129.51
129.39
128.82
128.42
127.53
127.29
125.21
123.11
122.15
121.68
115.05
114.84
114.50
114.27
77.32
77.00
76.68
29.68
15.51
2.66
200
190
180
170
160
150
140
130
120
110
100
90
80
70
60
50
40
30
20
10
ppm

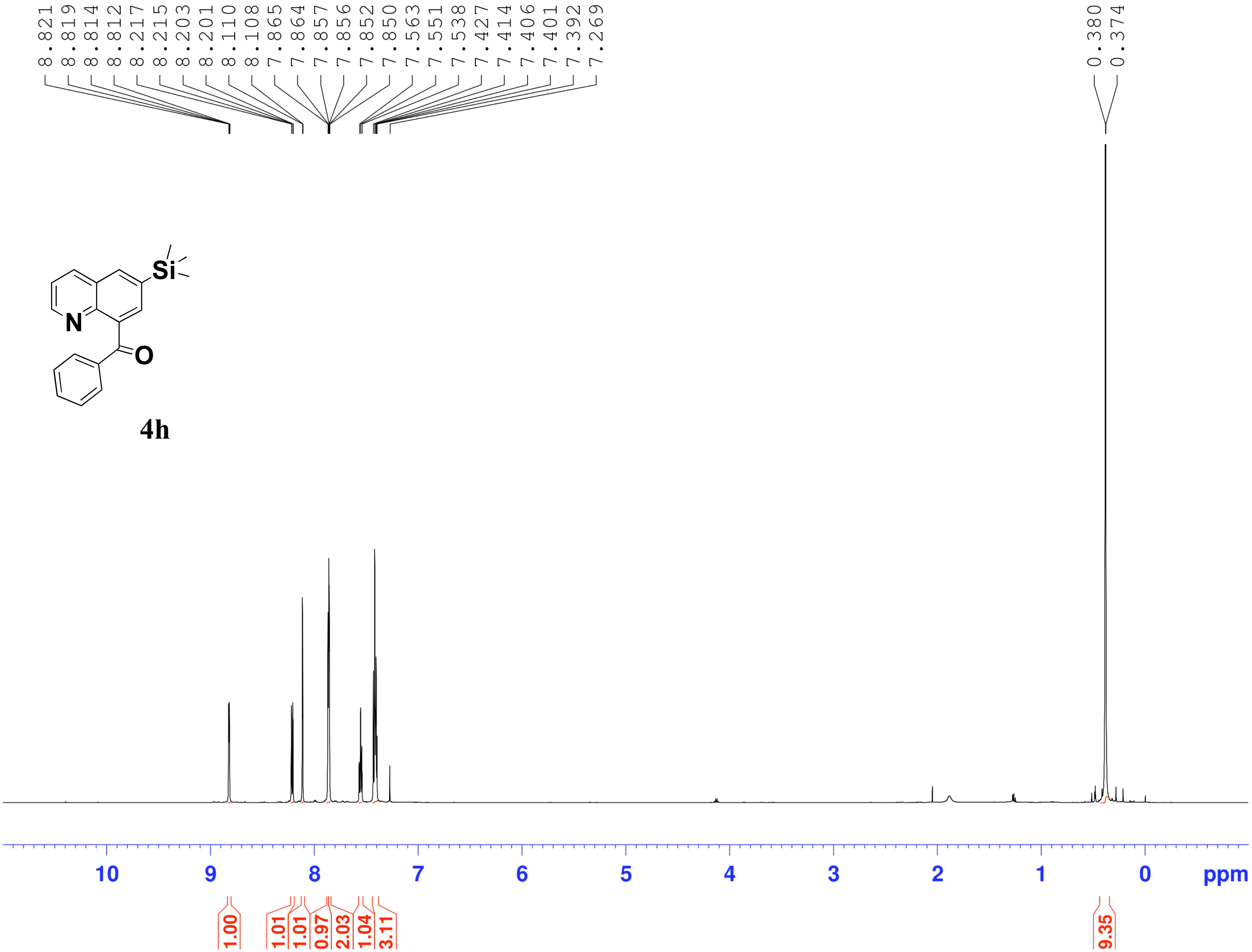

8.821
8.819
8.814
8.812
8.217
8.215
8.203
8.201
8.110
8.108
7.865
7.864
7.857
7.856
7.852
7.850
7.563
7.551
7.538
7.427
7.414
7.406
7.401
7.392
7.269
0.380
0.374
Si
N
O
10
9
8
7
6
5
4
3
2
1
0
ppm
1.00
1.01
1.01
0.97
2.03
1.04
3.11
9.35

4h

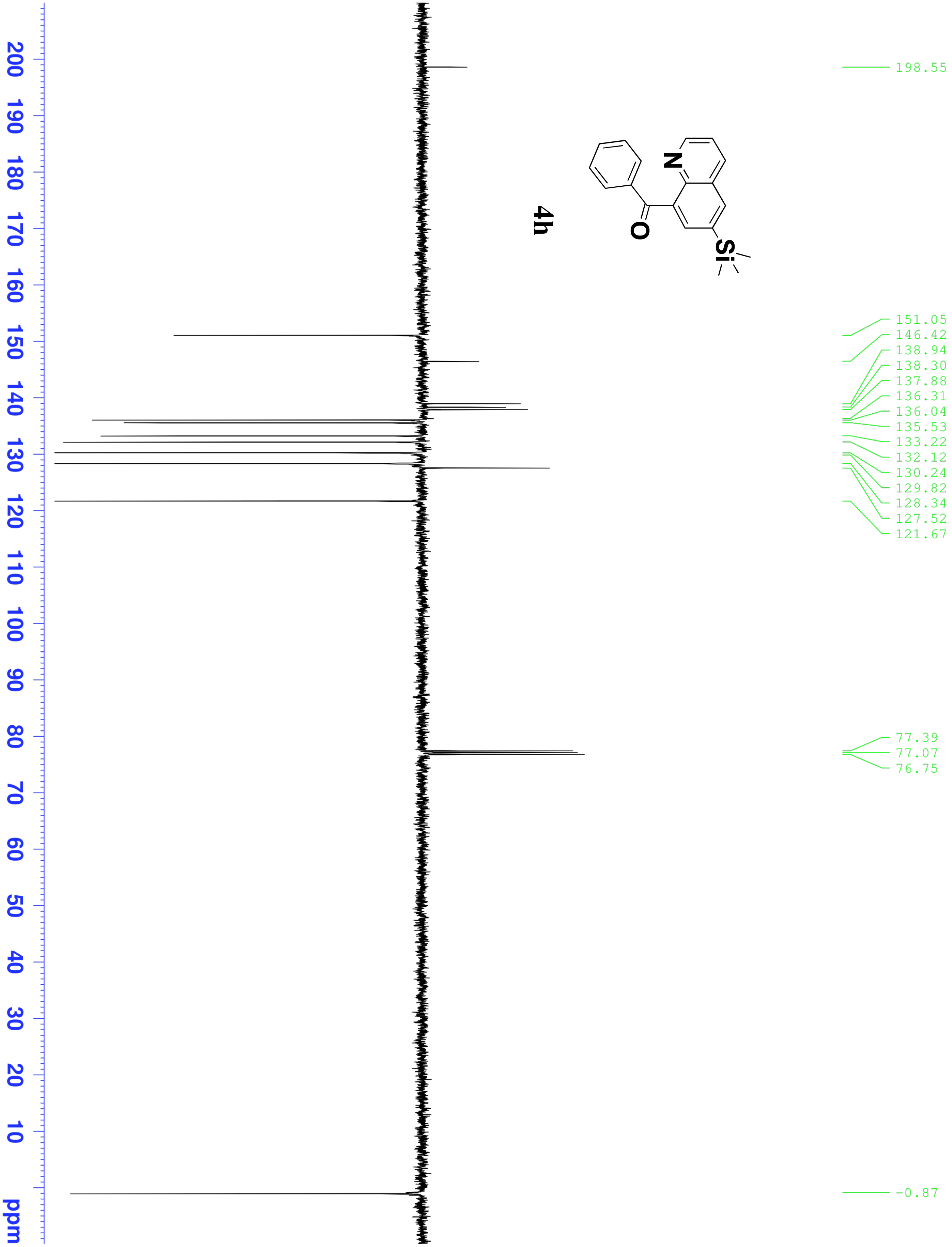

198.55
151.05
146.42
138.94
138.30
137.88
136.31
136.04
135.53
133.22
132.12
130.24
129.82
128.34
127.52
121.67
77.39
77.07
76.75
-0.87
4h
N
O
Si
ppm

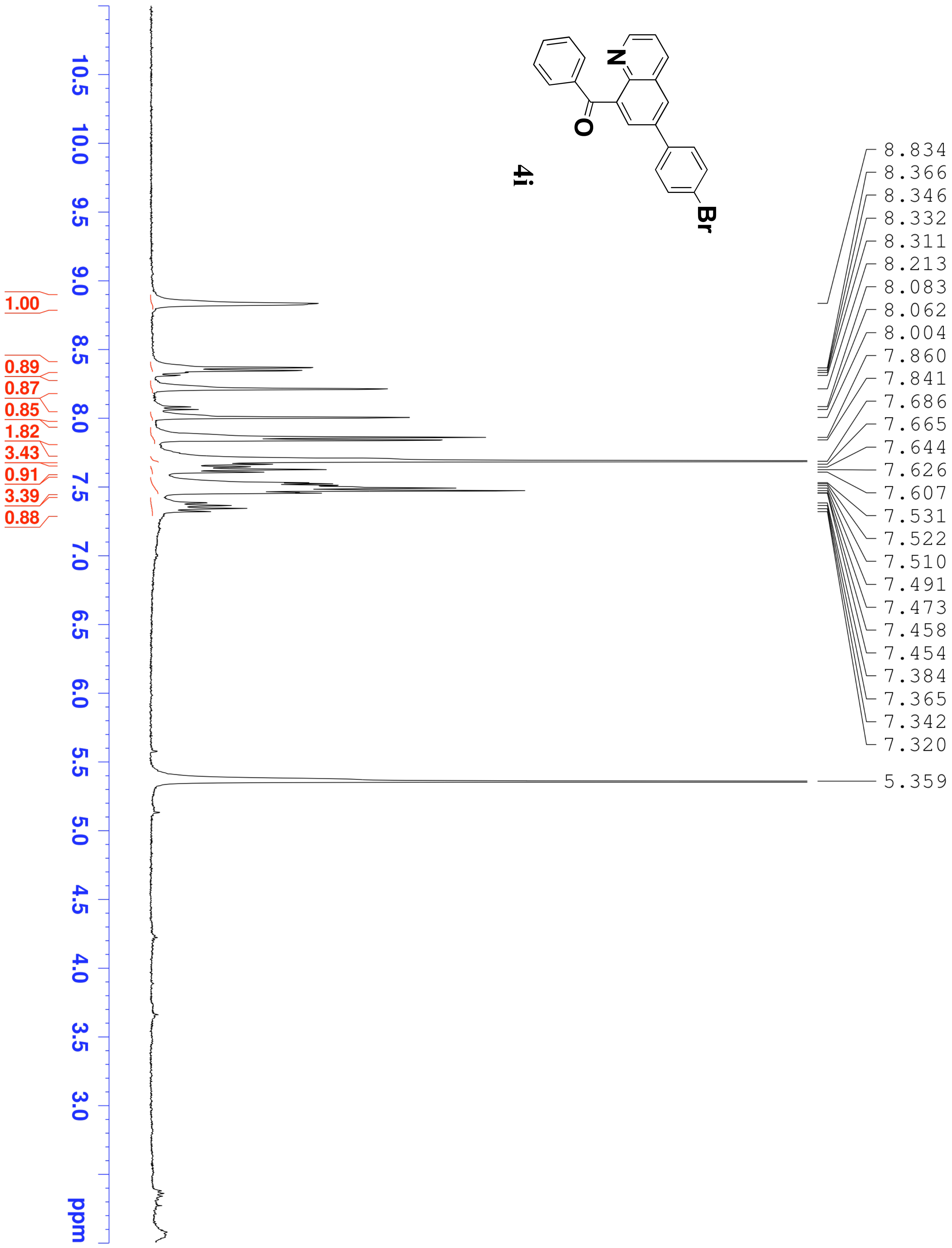
4i
N
O
Br
8.834
8.366
8.346
8.332
8.311
8.213
8.083
8.062
8.004
7.860
7.841
7.686
7.665
7.644
7.626
7.607
7.531
7.522
7.510
7.491
7.473
7.458
7.454
7.384
7.365
7.342
7.320
5.359
1.00
0.89
0.87
0.85
1.82
3.43
0.91
3.39
0.88
10.5
10.0
9.5
9.0
8.5
8.0
7.5
7.0
6.5
6.0
5.5
5.0
4.5
4.0
3.5
3.0
ppm

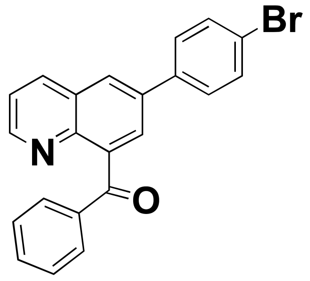

4i

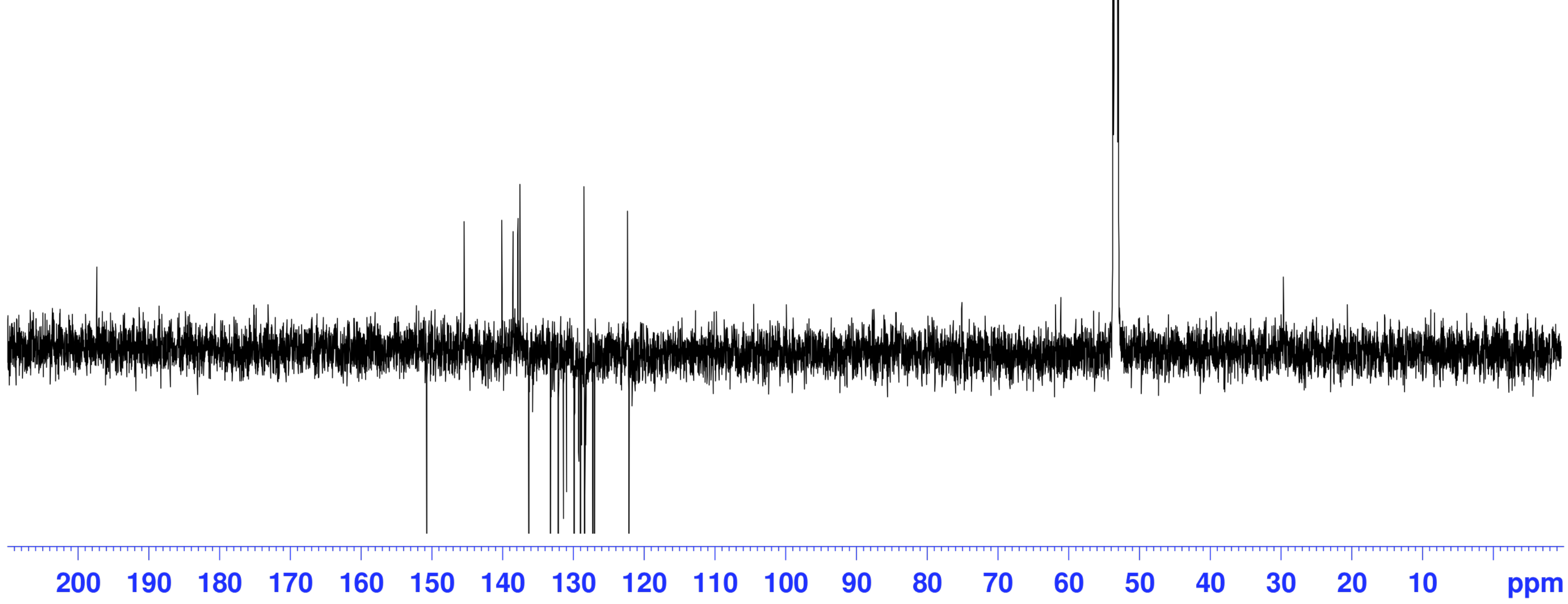

197.31
145.40
140.05
138.48
137.79
137.49
128.43
122.29
53.65
53.48
53.30
53.11
52.93
29.59
200
190
180
170
160
150
140
130
120
110
100
90
80
70
60
50
40
30
20
10
ppm

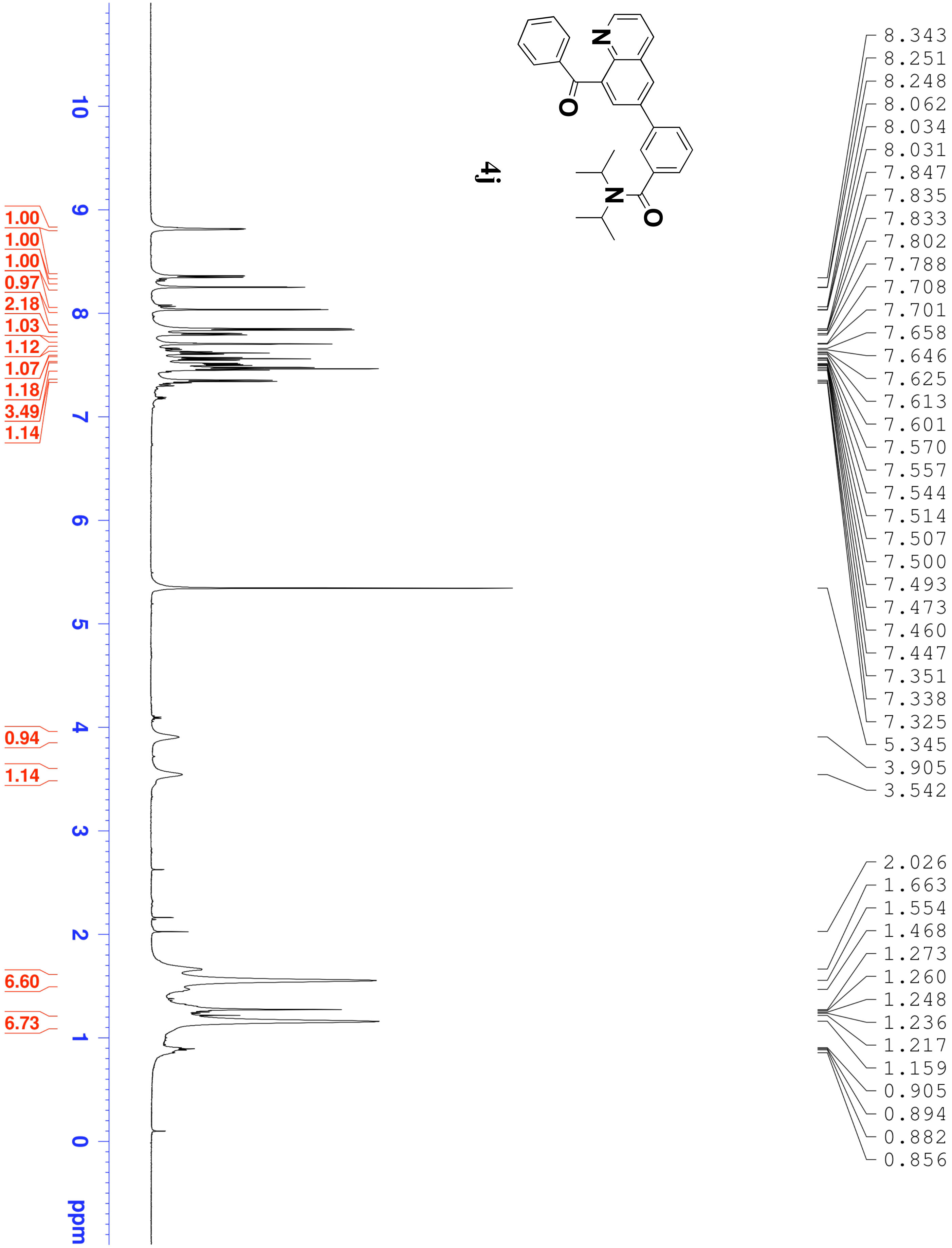
4j
8.343
8.251
8.248
8.062
8.034
8.031
7.847
7.835
7.833
7.802
7.788
7.708
7.701
7.658
7.646
7.625
7.613
7.601
7.570
7.557
7.544
7.514
7.507
7.500
7.493
7.473
7.460
7.447
7.351
7.338
7.325
5.345
3.905
3.542
2.026
1.663
1.554
1.468
1.273
1.260
1.248
1.236
1.217
1.159
0.905
0.894
0.882
0.856
1.00
1.00
1.00
0.97
2.18
1.03
1.12
1.07
1.18
3.49
1.14
0.94
1.14
6.60
6.73
10
9
8
7
6
5
4
3
2
1
0
ppm

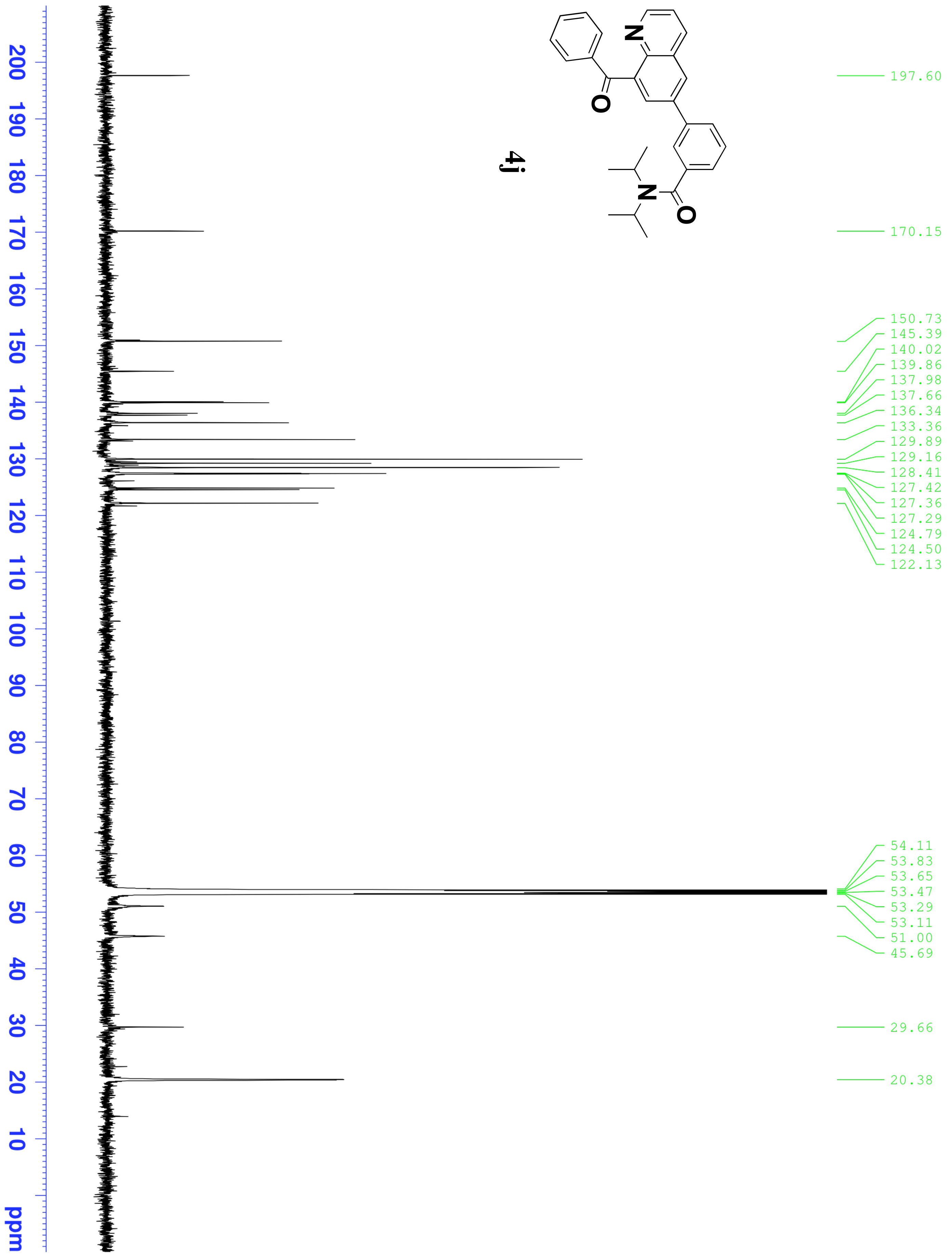
197.60
170.15
150.73
145.39
140.02
139.86
137.98
137.66
136.34
133.36
129.89
129.16
128.41
127.42
127.36
127.29
124.79
124.50
122.13
54.11
53.83
53.65
53.47
53.29
53.11
51.00
45.69
29.66
20.38
4j
ppm

References

1. Obika, S.; Kono, H.; Yasui, R.; Yanada, Y.; Takemoto, Y. *J. Org. Chem.* **2007**, 72, 4462-4468.

2. Dai, G.; Larock, R.C. *Org. Lett*. **2001**, 3, 4035-4038.

3. Dyker, G.; Stirner, W.; Henkel, G. *Chem Eur. J.* **2000**, 8, 1433-1441.

4. Barluenga, J.; Vasquez-Villa, H.; Ballesteros, A.; Gonzalez, J.M. *Org. Lett*., **2003**, 5, 4121-4123.

5. Asao, N.; Aikawa, A.; Yamammoto, Y. *J. Am. Chem. Soc*., **2004**, 126, 7458-7459.

6. Asao, N.; Takahashi, K.; Lee, S.; Kasahara, T.; Yamammoto, Y. *J. Am. Chem. Soc*., **2002**, 124, 12650-12651.
